# Palatal segment contributions to midfacial growth in three inbred mouse strains

**DOI:** 10.1101/2023.10.03.560703

**Published:** 2023-10-05

**Authors:** Ian C. Welsh, Maria E. Feiler, Danika Lipman, Isabel Mormile, Karissa Hansen, Christopher J. Percival

**Affiliations:** Program in Craniofacial Biology, Department of Orofacial Sciences and Department of Anatomy, University of California at San Francisco, San Francisco, California 94143, USA; Interdepartmental Doctoral Program in Anthropological Sciences, Stony Brook University, Stony Brook, NY 11790; Department of Cell Biology and Anatomy, University of Calgary; Interdepartmental Doctoral Program in Anthropological Sciences, Stony Brook University, Stony Brook, NY 11790; Program in Craniofacial Biology, Department of Orofacial Sciences and Department of Anatomy, University of California San Francisco, San Francisco, CA 94143; Department of Anthropology, Stony Brook University, Stony Brook NY 11794

**Keywords:** palatogenesis, secondary palate, rugae, inbred mouse strains, face elongation, RRID:IMSR_JAX:003715, RRID:IMSR_JAX:001976, RRID:IMSR_JAX:000664

## Abstract

Following facial prominence fusion, anterior-posterior (A-P) elongation of the palate is a critical aspect of palatogenesis and integrated midfacial elongation. Reciprocal epithelial-mesenchymal interactions drive secondary palate elongation and periodic signaling center formation within the rugae growth zone (RGZ). However, the relationship between RGZ dynamics and the morphogenetic behavior of underlying palatal bone mesenchymal precursors has remained enigmatic. Our results indicate that cellular activity at the RGZ simultaneously drives rugae formation and elongation of the maxillary bone primordium within the anterior secondary palate, which more than doubles in length prior to palatal shelf fusion. The first formed palatal ruga, found just posterior to the RGZ, represents a consistent morphological boundary between anterior and posterior secondary palate bone precursors, being found at the future maxillary-palatine suture. These results suggest a model where changes in RGZ-driven A-P growth of the anterior secondary palate may produce interspecies and intraspecies differences in facial prognathism and differences in the proportional contribution of palatal segment-associated bones to total palate length. An ontogenetic comparison of three inbred mouse strains indicated that while RGZ-driven growth of the anterior secondary palate is critical for early midfacial outgrowth, subtle strain-specific bony contributions to adult palate length are not present until after this initial palatal growth period. This multifaceted illustration of normal midfacial growth dynamics confirms a one-to-one relationship between palatal segments and upper jaw bones during the earliest stages of palatal growth, which may serve as the basis for evolutionary change in upper jaw morphology. Additionally, identified mouse strain-specific differences in palatal segment elongation provide a useful foundation for understanding the impact of background genetic effects on facial morphogenesis.

## Introduction

Morphological variation of the midfacial complex, which consists of the nose, upper jaw, cheek, and palate, is a defining aspect of both intra- and inter-specific differences in facial shape. Evolutionary differences in midfacial prognathism can be quantified as variation in upper jaw bone elongation and in the proportional contribution of specific bones to total upper jaw length. Species-specific differences in the size, shape, and growth trajectories of cranial neural crest (CNC)-derived facial prominences, the frontonasal process (FNP) and maxillary processes (MxP), are identifiable during the earliest phases of facial morphogenesis, prior to skeletal differentiation, often presaging interspecies differences in adult facial shape (Selleri and Rijli, 2023). Notably, after initial facial prominence fusion, early facial growth often includes substantial anterior-posterior (A-P) midfacial elongation, leading to variable degrees of midfacial prognathism (Young et al., 2014); possibly based on the balance of CNC progenitor self-renewal versus osteoblast differentiation (Hall et al., 2014; Morris and Abzhanov, 2021; Schneider, 2015). Although characteristic interspecies differences in craniofacial morphology are often present at birth (Ponce de León and Zollikofer, 2001; Zumpano and Richtsmeier, 2003; Cobb and O’Higgins, 2004; Singleton, 2012; Smith et al., 2015; Woronowicz and Schneider, 2019; Kundrát et al., 2020), postnatal bone deposition and remodeling are also required to produce the highly differentiated adult morphology, which often includes more extreme midfacial prognathism (Enlow and Bang, 1965; Sarnat, 1997; Cobb and O’Higgins, 2004; Leigh, 2006; Martinez-Maza et al., 2013; Maga, 2016). Thus, both embryonic and postnatal developmental processes play a role in determining adult upper jaw size and shape.

**Figure 1 –:**
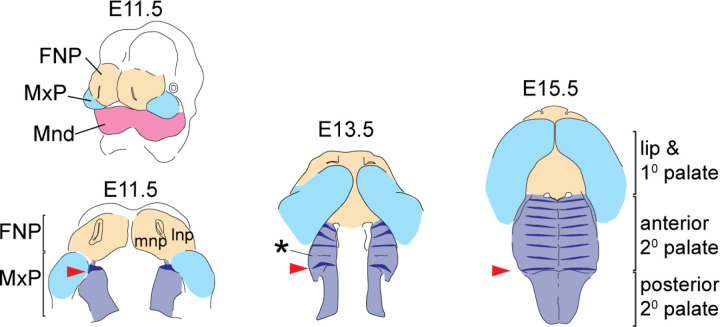
Tissue origins of the midfacial complex and rugae position during secondary palate morphogenesis – The upper and lower jaws are formed from the frontonasal process (FNP - tan), and branchial arch 1 derived maxillary and mandibular processes (MxP - pale blue and Mnd – magenta, respectively). From E11.5 to E15.5, outgrowth and fusion of the medial nasal process (mnp) with the superficial portion of the MxP frame out the lip and primary (1°) palate, while the A-P elongation and medially directed growth of the palatal shelves from the internal portion of the MxP gives rise to the secondary palate (2° pal – light purple). *Shh* expression (dark blue) highlights the dynamics of rugae formation and illustrates regional expansion of the anterior secondary palate. At E11.5, ruga 1 (red arrowheads) forms at the anterior extent of the nascent palatal shelf and subsequently defines the caudal end of the rugae growth zone (asterisk) where new rugae form prior to being displaced anteriorly. Additional abbreviation: lateral nasal process (lnp).

Basic facial shape is the result of facial prominence outgrowth and a series of fusion events, which occur between embryonic day (E) 10 and E15 in mice (Fig. 1). Initial fusion between the medial nasal processes (mnp) of the FNP and the anterior MxP gives rise to the primary palate and lip while also producing a unified upper jaw from previously separated tissues. Disproportional growth of FNP or MxP prior to fusion can result in cleft lip and palate, leading to reduced evolutionary fitness. Conversely, differential growth of FNP and MxP derived tissues after facial prominence fusion is a major basis for evolutionary differences in midfacial elongation; where facial elongation is driven primarily by MxP growth in mammals and by FNP growth in birds (Young et al., 2014).

Soon after primary palate fusion at E11.5 in mice, secondary palate morphogenesis begins through outgrowth of the nascent palatal shelf along the medial aspect of the MxP. Significant palatal growth along the A-P axis is accompanied by vertical growth of the palatal shelves prior to their elevation and medial fusion dorsal to the tongue, after which the palatal shelf separates the oral and nasal cavities (Bush and Jiang, 2012; Hammond and Dixon, 2022). A lack of coordination between the multiple growth axes of MxP-derived tissues can prevent secondary palate closure even when the palatal shelves maintain competency to fuse.

The mammalian palate is composed of three major longitudinal segments called the primary palate, anterior secondary palate, and posterior secondary palate. The boundary between primary palate and anterior secondary palate is the posterior edge of the fused mnps. The A-P organization of the secondary palate can be defined by various criteria including the presence of boney versus muscular tissue (i.e. the hard versus soft palate), modes of palatal shelf elevation and closure, and molecular differences in tissue patterning and cell signaling competence (Hilliard et al., 2005; Yu and Ornitz, 2011; Hammond and Dixon, 2022). We use mutually exclusive patterns of gene expression and the position of epithelial appendages called rugae to define the boundary between anterior secondary and posterior secondary palate, as described below.

From a morphogenetic regulatory perspective, the spatial expression of cell type specific transcription factors (TFs) and intercellular signaling molecules is particularly informative to understanding A-P organization of palate growth. For example, the CNC-derived mesenchymal expression of *Msx1* and *Shox2* is exclusive to the anterior secondary palate during palate morphogenesis, while *Barx1* and *Tbx22* expression is exclusive to the posterior secondary palate (Hilliard et al., 2005; Okano et al., 2006; Welsh et al., 2018). Mutation of *Shox2* or *Tbx22* results in midline clefts of the anterior or posterior palate, respectively (Yu et al., 2005; Pauws et al., 2009), although normal expression of these factors is maintained in genetically engineered mice with severe segmental growth defects (Welsh et al., 2018). The A-P expression boundary of these and other factors is found at the first formed palatal ruga (Hilliard et al., 2005; Li and Ding, 2007; Welsh et al., 2007; Welsh and O’Brien, 2009; Welsh et al., 2018; Hammond and Dixon, 2022), one of multiple parallel epithelial thickenings on the anterior secondary palate.

The first ruga (ruga 1 numbered according to (Welsh et al., 2007; Welsh and O’Brien, 2009, although see Pantalacci et al., 2008) forms at E11.5 on the anterior extent of the nascent secondary palate (Fig. 1). Remaining rugae form sequentially within the rugae growth zone (RGZ) located just anterior to ruga 1 (Welsh and O’Brien, 2009), via a Turing type mechanism (Economou et al., 2012; Kawasaki et al., 2018). Rugae are centers of Sonic Hedgehog signaling (SHH) that is critical for anterior secondary palatal patterning and A-P growth. As the secondary palate completes midline fusion at E15.5, the last formed ruga appears adjacent to ruga 1 in the middle palate while each precedingly formed ruga is found at an increasingly anterior position along the anterior secondary palate.

Based upon the dynamics of rugae formation, palate growth, and patterning gene expression, Pantalacci (et al., 2008) hypothesized that ruga 1 represents a distinct morphological boundary related to either the future hard versus soft palate boundary or the maxillary-palatine suture. However, the dynamics of rugae formation and A-P patterns of molecular and morphological variation in the secondary palate have not been explicitly defined in relation to the earliest stages of facial bone development. One goal of our research was to further clarify the degree to which early ontogenetic processes in the palate are coordinated with skeletal differentiation. To address this knowledge gap, we defined the anatomical and molecular relationship of the anterior and posterior secondary palate during anterior secondary palate outgrowth with reference to boundary ruga 1, the rugae forming RGZ, and the broader context of midfacial anatomical structures. We systematically mapped the initial specification and early differentiation of osteoblasts relative to this anatomically defined segmental organization across a developmental time course spanning mouse secondary palate morphogenesis and upper jaw outgrowth (E11.5-E15.5). Visualization of molecular markers during the earliest phases of palatal morphogenesis reveals the segmental origins of upper jaw bone formation and a previously unappreciated coupling of maxillary formation to rugae morphogenesis during expansion of the anterior secondary palate.

Given that 1) there is strong early one-to-one association between upper jaw bones and the three major palatal segments (primary, anterior secondary, posterior secondary), 2) morphogenic and differentiative dynamics are distinct along the A-P axis of the elongating midface, and 3) complex embryonic processes at cellular and tissue levels are often critical for the production of typical facial shape (Hilliard et al., 2005; Kaucka et al., 2018; Yuan et al., 2020), early differences in the longitudinal growth of the three palatal segments might underlie inter- and intraspecies differences in upper jaw morphology. Specifically, we hypothesized that genetically based differences in palate segment-specific A-P outgrowth would explain previously identified variation in the contribution of the premaxilla, maxilla, and palatine bones to adult upper jaw morphology of inbred mouse strains (Percival et al., 2016). For example, a minor segment-specific reduction in the growth rate of the anterior secondary palate between E11.5 and E15.5 could result in a relatively short anterior secondary palate and relatively short adult maxillary bone, as identified within the C57BL/6J (C57; RRID:IMSR_JAX:000664) strain.

To test our hypothesis and shed light on the overall developmental basis of adult palatal morphology, we measured the facial morphology of an ontogenetic series of common inbred strains of mice between E11 and E15, one day after birth, and in adults (8–12 weeks old). The C57 and NOD/ShiLtJ (NOD; RRID:IMSR_JAX:001976) inbred strains were chosen because C57 adults have a relatively long premaxilla and NOD adults have a relatively long maxilla (Percival et al., 2016). The PWK/PhJ (PWK; RRID:IMSR_JAX:003715) wild-derived inbred strain was chosen as a mouse with a smaller adult body size, a relatively long maxilla compared to the premaxilla, and a relatively long upper jaw for its body size when compared to the other strains (Percival et al., 2016). If variation in early A-P elongation of longitudinal palatal shelf segments is the causal basis for strain specific differences in upper jaw morphology, we predicted that C57 mice would have a relatively long primary palate and a relatively short anterior secondary palate by E15 and that the other two strains would display relatively long anterior secondary palates at the same developmental stage.

Understanding how genetically based differences in early craniofacial development produce morphological variation within model organisms is an important step in determining how these developmental processes can be modified to produce evolutionary differences in upper jaw morphology (Boughner et al., 2008; Hallgrímsson et al., 2009; Fish, 2019; Morris and Abzhanov, 2021), including facial length and the relative contribution of specific bones to the upper jaw. The subtle strain-associated differences in palatal morphology we report are reminiscent of brachycephalic versus dolichocephalic dog breeds and species-level differences between primates, including the reduced facial length of humans compared to other apes and the increased facial length of baboons compared to other cercopithecines. Overall, our multifaceted analysis of ontogenetic trends in palatal and facial elongation provides a novel contextual framework and developmental perspective within which to evaluate the impact of both non-pathogenic as well as pathogenic genetic differences on midfacial growth and differentiation.

## Methods

### Specimen and Image Acquisition

Animal breeding, specimen collection, and tissue fixation were performed in accordance with the protocols of the University of California, San Francisco Institutional Animal Care and Use Committee under protocol approval number AN192776–01F. Mice were socially housed under a twelve-hour light-dark cycle with food and water *ad libitum*. Additional enrichment was provided when single housing was required for breeding purposes. Mice were euthanized by CO_2_ inhalation followed by cervical dislocation or decapitation. Embryos of C57BL/6J (RRID:IMSR_JAX:000664; C57), NOD/ShiLtJ (RRID:IMSR_JAX:001976; NOD), and PWK/PhJ (RRID:IMSR_JAX:003715; PWK) strains (Jackson Labs, Bar Harbor, ME) were collected between gestational days E11.5 and E15.5, as determined from copulatory plug occurrence. Postnatal day one (P1) C57 (n=10) and NOD (n=7) specimens were collected one day after birth. Embryo and P1 specimens were fixed in 4% PFA and stored in 1x PBS for micro-computed tomography (μCT) imaging or dehydrated through a graded PBST-MeOH series and stored at −20°C until use for in situ hybridization.

Specimens for µCT scanning were received, stored, and imaged at the University of Calgary in accordance with the protocols of the University of Calgary Institutional Care and Use Committee under approval number AC13–0268. After approximately an hour of soaking in Cysto-Conray II (Liebel-Flarsheim Canada), µCT images of embryo heads were acquired with a Scanco µ35 with 45kV/177µA for images of 0.012 mm^3^ voxel size. µCT images of P1 heads were acquired similarly, but with 0.021 mm^3^ voxel size. Photographs of embryo hindlimb buds were collected using a dissecting microscope for developmental age estimation. Adult specimens were previously collected and µCT imaged, as described by Percival et al., 2016.

### In Situ Hybridization

In situ hybridization was performed as described in Welsh et al., 2018. cDNA probes for *Phospho1* (nucleotides 274–1739 of RefSeq NM_153104), *Runx2* (nucleotides 1275–2704 of RefSeq NM_001271627), *Shh* (full length cDNA clone from A. McMahon, University of Southern California, Los Angeles CA, USA), *Shox2* (nucleotides 870–1603 of RefSeq NM_130458), *Sp7* (nucleotides 431–1715 of RefSeq NM_130458), *Tbx22* (nucleotides 307–1547 of RefSeq NM_013665) were linearized and in vitro transcribed to label with either digoxygenin (DIG) or dintrophenol (DNP). Colorimetric detection of probes used BM purple (dark blue), BCIP (teal), or MagentaPhos (magenta).

### DMDD HREM Data

High resolution episcopic microscopy (HREM) data (Geyer et al., 2009) was generated as part of the deciphering mechanisms of developmental disorders (DMDD) program (Wilson et al., 2016) and is available from the BioImage Archive at: https://www.ebi.ac.uk/biostudies/bioimages/studies/S-BIAD490?query=DMDD

### Embryonic Palatal Segment Growth Trajectories

#### Developmental Age Estimation

Given that common mouse strains vary in gestation length and there is variation in developmental timing within litters, it was necessary to standardize our cross-strain morphometric analysis by embryonic developmental age rather than gestational age. Developmental age was estimated for each µCT scanned embryonic specimen using eMOSS, an application that predicts developmental age from hindlimb bud outlines, based on a previous analysis of C57 mice (Musy et al., 2018). The resulting limb-based estimates of developmental age were reported as days since conception, up to two decimal places. We combined similar developmental age estimates into developmental age categories so that specimens were assigned to a whole- or half-day developmental age within 0.25 days of their initial eMOSS estimate (Table 1). For example, an eMOSS estimated E12.20 specimen was assigned to the E12.0 limb-based developmental age category.

Additional error may be expected when estimating the developmental age of NOD and PWK embryos using a predictive model built on C57 strain limb bud ontogeny, given the possibility of differences in the correlation of limb and head development between strains. Therefore, we also utilized the first principal component of embryonic head shape variation (PC1), based on the landmark analysis described below, as a proxy for developmental age in some analyses. PC1 score consistently tracked ontogenetic change in facial and palatal shape across strains (see Results).

#### Anatomical Landmark Collection

**Figure 2 –:**
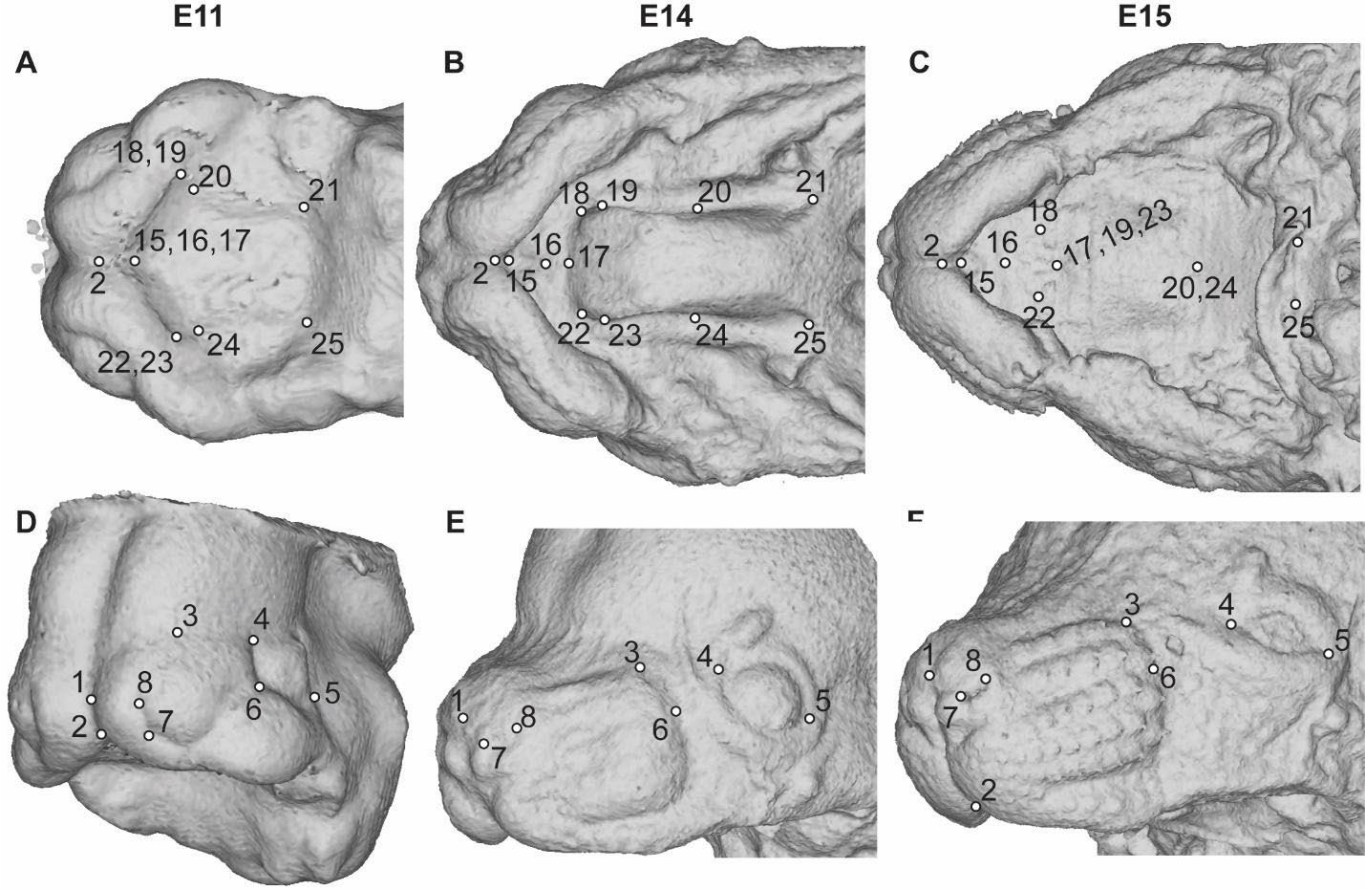
Anatomically homologous epithelial landmarks collected for the quantification of midfacial and palatal shape across limb based developmental stages, from (a-c) palatal and (d-f) oblique facial views. See also Supporting Table 1.

All embryo facial and palate landmarks were collected within Meshlab (Cignoni et al., 2008) on minimum threshold-based superficial tissue surfaces (downsampled x2) produced from the µCT images. Landmarks that could be identified consistently in anatomically homologous positions across the embryonic period of facial growth were chosen around the nose and whisker region, the eyes, and along the palate (Fig. 2, Supporting Table 1). Care was taken to define epithelial tissue palatal landmarks in embryos that could be associated with the bony morphology of the P1 and adult mouse upper jaw.

#### Prenatal Geometric Morphometric Analysis

A Procrustes superimposition-based geometric morphometric analysis was used to quantify the ontogenetic shape change of the palate and face for the embryonic C57 sample and deviations of the NOD and PWK strains from this C57 baseline. Embryonic specimens with limb-based developmental age estimates between E11 and E15 were included in the embryonic shape analysis (Table 1). We performed a geometric morphometric analysis of facial landmarks using *geomorph* (Adams et al., 2020) and RRPP (Collyer and Adams, 2018) libraries in R Statistical Software (R Core Team, 2021). We first performed a generalized Procrustes analysis (GPA) to align specimen landmark sets by translating, scaling, and rotating their landmark coordinates (reviewed by Zelditch et al., 2012). Embryonic specimen shape analyses were completed using the symmetric component of Procrustes-aligned specimen landmark coordinate variation, as we assumed that most bilateral shape differences between the left and right sides of a specimen’s face are due to random effects associated with developmental noise and tissue fixation (Palmer and Strobeck, 1986). Thus, symmetrized landmark coordinate data were interpreted to represent the facial shape defined by a given inbred strain genotype.

The mean facial shape of specimens within each developmental age category were estimated for each strain. Differences between the mean shapes of a subset of developmental age categories within the C57 sample were plotted to illustrate typical facial/palatal shape growth trajectories during this important period of secondary palate elongation and midline fusion.

A principal components analysis (PCA) was completed to identify the major axes of shape covariation across the embryonic sample. As with most PCA of ontogenetic series, the first principal component (PC1) was strongly associated with overall specimen size and specimen developmental age. Therefore, we treated it as a proxy for ontogenetic growth and developmental age. C57-specific segmented linear regressions represented a baseline of overall facial/palatal shape change to which the NOD and PWK specimens were compared. Due to clear inflection points in the slopes of embryonic specimen PC scores when plotted against PC1, segmented linear regressions were completed separately for PC2, PC3, and PC4 versus PC1 values for the C57 sample.

After removing three NOD specimens with PC1 scores that were substantially lower than the minimum C57 PC1 score that the C57 regression was based on, we predicted PC2, PC3, and PC4 scores from each C57, NOD, and PWK specimen’s PC1 score based on the C57 baseline segmented regressions. Then we calculated the PC score residuals of each specimen as the difference between predicted and measured PC scores. We completed Wilcoxon rank sum tests to identify significant genotypic differences for PC2, PC3, and PC4 residual values. A significant difference in mean PC score residual values between strains was interpreted as a significant difference in the ontogeny of facial shape for that pair of strains.

#### Prenatal Palate Segment Length

The anterior-posterior length of the three major palatal segments (i.e., primary palate, anterior secondary palate, and posterior secondary palate) were estimated from landmark coordinates without any superimposition or scaling. To estimate the length of these segments along the anterior-posterior axis of the palate, the mean position of bilateral pairs of palatal landmarks were calculated (i.e., the midline projection of the bilateral landmarks). Primary palate, anterior secondary palate, and posterior secondary palate lengths were calculated in millimeters from these landmark positions. Proportional palatal segment lengths were calculated as the length of a single segment divided by the sum of all three segment lengths for a given specimen. LM15 represented the midline anterior extent of the primary palate, the midpoint between LM18 and LM22 represented the border between primary palate and the anterior secondary palate, the midpoint between LM20 and LM24 represented the border between the anterior and posterior secondary palate, the midpoint between LM21 and LM25 represented the posterior boundary of the posterior secondary palate.

Small strain-specific sample sizes at multiple limb-based developmental ages meant it was not possible to statistically test whether the genetic strains had different proportional palatal segment contributions at single developmental ages. Instead, separate second-degree polynomial regressions of proportional palate segment lengths versus specimen facial shape PC1 score were calculated for the C57 embryos. Because PC1 score was considered a proxy for developmental age, these regressions define ontogenetic trajectories of palatal segment contributions to overall palatal length, which we used as a baseline for comparison with NOD and PWK proportional segment lengths. After removing three NOD specimens with PC1 scores that were substantially lower than the minimum C57 PC1 score, we predicted the proportional length of each palatal segment for all specimens based on the C57 regressions. The residual differences in proportional palatal segment lengths between predicted and measured values were compared across the three strains using Wilcoxon rank sum tests. A significant difference in mean residual values was interpreted to indicate a generally higher or lower proportional contribution of a given palate segment to overall palatal length across the embryonic period being measured.

### Postnatal Palate Segment Length Comparisons

To determine if embryonic differences in proportional palate segment length remained consistent postnatally and to investigate the degree of correspondence between surface epithelial and skeletal measures of palatal variation, postnatal specimen landmarks were collected on minimum threshold-based surfaces produced from *μ*CT scans. Superficial epithelial surfaces of P1 specimens were produced using Amira (downsampled x2), and epithelial landmarks (Supporting Table 1) were collected within Meshlab (Cignoni et al., 2008).

Minimum threshold-based skeletal surfaces of P1 and adult specimens were produced using 3D Slicer (Fedorov et al., 2012) after Gaussian blur image filtering (sigma set to 0.01 for P1; sigma set to 0.02 for adult). We identified skeletal anatomical landmarks that most closely matched the palate segment landmarks defined on surface epithelium (Supporting Table 2). Landmarks were collected for C57 (n=10) and NOD (n=8) P1 specimens and for C57 (n=20), NOD (n=18), and PWK (n=18) adult specimens. Midline palate segment lengths and associated upper jaw bone lengths were measured using landmark coordinates without scaling or superimposition. Midline points were calculated from pairs of bilateral landmarks defining each palate segment boundary, midline projected palate segment lengths were estimated for each specimen, and total palate length was calculated as the sum of the three midline palate segment lengths. Proportional palate segment lengths were calculated as individual segment lengths divided by total palate length. Wilcoxon rank sum tests were used to identify significant differences in proportional palate segment lengths between strains within single age and tissue type categories.

## Results

### Defining an Anatomical and Molecular Reference Framework of Secondary Palate A-P Growth

**Figure 3 –:**
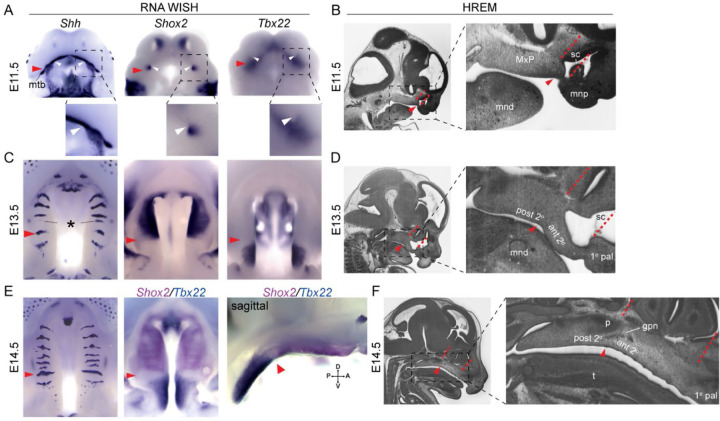
A-P molecular heterogeneity and anatomical relationships during secondary palate morphogenesis. (a, c, e) Whole mount in situ hybridization for *Shh*, *Shox2*, and *Tbx22* expression, and (b, d, f) sagittal sections of wildtype embryos imaged with high resolution episcopic microscopy to provide histological resolution of the primary (1° pal), anterior secondary (ant 2°), and posterior secondary palate (post 2°) relative to surrounding facial structure during midfacial outgrowth at (a, b) E11.5 (c, d) E13.5, and (e, f) E14.5. Throughout midfacial outgrowth, ruga 1 (red arrowheads) marks the shared A-P expression boundary of *Shox2* and *Tbx22* in the anterior and posterior secondary palate, respectively. Double label WISH for *Shox2* (magenta) and *Tbx22* (blue) at E14.5 highlights mutually exclusive anterior and posterior expression domains organized relative to ruga 1 (oral view left, sagittal view right). White arrowheads mark the choanae, black asterisk marks the RGZ. Red dashed lines mark the primary-secondary palate junction and posterior wall of the nasal capsule to highlight coordinated elongation of the anterior secondary palate and overlying sinus cavity (sc). Regions in dashed boxes of HREM images shown enlarged to the right. Additional abbreviations: mandible (mnd), molar tooth bud (mtb), maxillary process (MxP), medial nasal process (mnp), tongue (t), greater palatine nerve (gpn), palatine (p).

We defined the anatomical relationship between the boundary ruga 1, the surrounding midfacial anatomy, and the A-P expression of genes critical for palatal shelf patterning as a foundation for assessing rugae morphogenesis and segmental growth dynamics of the secondary palate. A-P secondary palate elongation was defined with reference to the first ruga’s position at the boundary between the anterior and posterior secondary palate and the A-P expression of genes critical for palatal shelf patterning, including *Shox2* and *Tbx22*. Ruga 1 formed within the MxP at the anterior extreme of the nascent secondary palate (Fig. 3, red arrowheads). Expression of *Shox2* overlapped the medial portion of the *Shh* expression domain in forming ruga 1, while *Tbx22* palatal shelf expression was found just posterior to ruga 1. Sequential rugae formation occurred anterior to ruga 1 within the RGZ and accompanied expansion of the anterior secondary palate. *Shox2* expression remained restricted to the rugae-bearing anterior secondary palate, while *Tbx22* expression was restricted posterior to ruga 1 throughout this critical period of secondary palate elongation (Fig. 3a,c,e).

Close relationships between different anatomical domains underlying midfacial outgrowth are found centered at ruga 1, which is at the boundary between the anterior and posterior secondary palate. Elongation of the anterior secondary palate is coordinated with the formation of the overlying sinus cavity, and disrupted morphogenesis of either structure can impact the other (Pauws et al., 2009; Kaucka et al., 2018; Welsh et al., 2018). We found that ruga 1 formed adjacent to the choanae, the posterior openings of the forming sinus cavity (Fig. 3a, white arrowheads). Expansion of the secondary palate anterior to ruga 1 between E11.5 and E15.5 anteriorly displaced the primary palate and external nares. Ruga 1 and the posterior wall of the nasal capsule remained approximately in the same coronal plane during elongation of the anterior secondary palate and nasal capsule (Fig.3b,d,f), suggesting that they are positioned at a proximal source of directional growth. Ruga 1 was also coincident with a gross morphological inflection point of the palatal tissue where the anterior secondary palate and overlying sinus cavity exhibited a more inferiorly accentuated angle relative to the cranial base than the posterior secondary palate did (Fig. 3d-e, Supporting Fig. 1). Lastly, between E12.0 and E12.5, the greater palatine nerve and artery were found entering the palatal shelves from a location immediately dorsal to ruga 1. This spatial relationship was maintained until the greater palatine foramen forms prior to E15.5 by the articulation of maxilla and palatine bones (Fig. 3f, Supporting Fig. 1). These anatomical relationships collectively demonstrated that ruga 1 represents an important stable anatomical boundary and point of reference within the context of early midfacial outgrowth and segmental organization of the palate.

**Figure 4 –:**
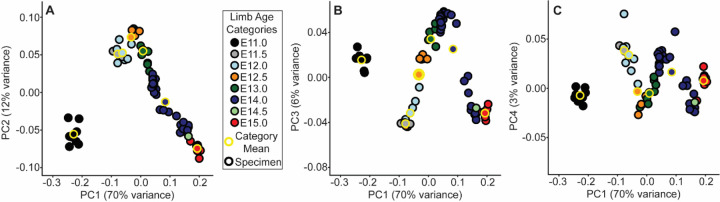
Major axes of embryonic facial shape variation - C57 embryo specimens plotted along the (a) first and second principal component axes of shape (i.e., PC1 and PC2), (b) along PC1 and PC3, and (c) along PC1 and PC4. Circle color indicates the limb based developmental age categories of each specimen, with the yellow bordered circles indicating the mean PC scores for the C57 mice of that age category. The proportions of facial shape variance associated with each principal component are provided. The principal component axes were estimated from the full sample of E11-E15 developmental age embryos, including C57, NOD, and PWK samples, although only C57 samples are plotted in this figure. The same plot, but incorporating all three inbred strains is available as Supporting Figure 2.

We conducted a complementary 3D morphometric analysis of epithelial landmarks across the midface, including several defined by the palatal anatomy described above, to quantify the integrated morphogenetic processes occurring across the facial region during this critical early developmental period. The first major principal axis of facial shape variation (PC1) for all limb bud staged E11-E15 specimens represented the majority of shape variance present in the sample (70%) and was associated with general ontogenetic growth during this time window, as illustrated for the C57 sample (Fig. 4) and for all three inbred strains (Supporting Fig. 2). Additional principal axes of variation suggested shifts in the direction of ontogenetic shape changes over developmental time. For example, the PC2 scores of C57 embryos increased from E11 to E12.5, but then decreased again from E12.5 to E15 (Fig. 4a). The direction of shape change shifted twice along PC3 (Fig. 4b) and three times along PC4 (Fig. 4c).

**Figure 5 –:**
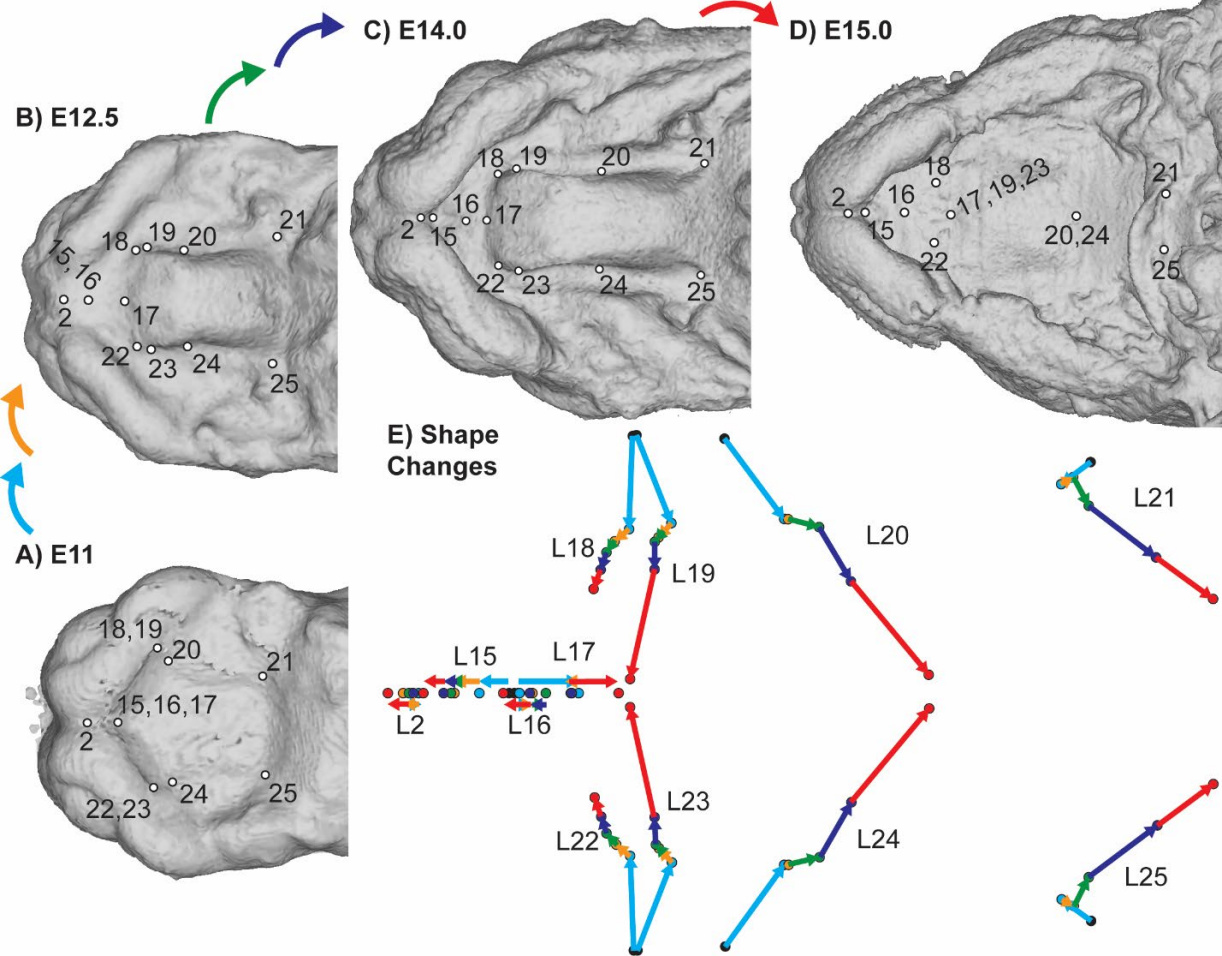
C57 palatal landmark growth trajectories – The positions of palatal landmarks are identified on representative C57 specimens at limb-derived developmental ages (a) E11, (b) E12.5, (c) E14, and (d) E15. (e) The average palatal landmark positions are plotted for each developmental age category that had more than one C57 specimen. These landmark positions represent palatal shape after the removal of overall facial scale. The arrows indicate the trajectory of shape change for each landmark between ages. Black=E11; Light Blue = E12; Orange = E12.5; Green = E13; Dark Blue = E14; Red = E15.

Average developmental age category shape differences were illustrated as landmark specific ontogenetic shifts in palate shape (Fig. 5) and overall midfacial shape (Supporting Fig. 3). Mouse embryos grew substantially between limb-based developmental ages E11 and E15, including increased facial size and oral cavity length. Within C57 mice at E11, the mnp and the MxP fused to create the primary palate, while the cell populations that will give rise to the posterior secondary palate were already visible. Compared to E11, E12.5 specimens had a relatively longer primary palate and a relatively short distance between the left and right secondary palatal shelves. After E12.5, secondary palate length (relative to overall facial size) increased noticeably with each developmental stage. As the specimens approached E15, the most substantial changes in palatal shape were related to 1) the previously described A-P elongation of the secondary palate and 2) the medial growth and ultimate fusion of the palatal shelves.

Between developmental stages E11 and E15, each of the three major palatal segments grew in length, but the anterior secondary palate grew proportionally more than the primary palate and posterior secondary palate (Figs. 3 & 5). Midline epithelial measurements of C57 embryos soon after the start of A-P palatal elongation indicated that the primary palate represented ~20% of the total palate length, the anterior secondary palate ~20%, and the posterior secondary palate ~60%. In contrast, by the time of midline palatal fusion at E15.5, the primary palate represented ~25% of palate length, the anterior secondary palate represented >40%, while the posterior secondary palate represented <35%. Therefore, between E11 and E15, the proportional contribution of the primary palate increased by a quarter, while posterior secondary palate contribution decreased by nearly a third. Strikingly, the proportional contribution of the anterior secondary palate doubled during this period.

### Segmental Differences in Skeletal Specification, Differentiation, and Growth During Secondary Palate Morphogenesis

Cellular changes in gene expression represent one of the most proximal steps in the morphogenetic cascade of osteogenic differentiation and bone formation. We therefore sought to evaluate the earliest stages of midfacial skeleton specification and differentiation in relation to the pattern of segmental growth described above. We analyzed the expression of *Runx2*, *Sp7*, and *Phospho1* using whole mount in situ hybridization (WISH) on mouse embryos from E11.5 to E15.5, a developmental time course spanning the emergence of the secondary palate until midline palatal shelf fusion. These molecular markers were chosen because *Runx2* expression overlaps the initial specification of osteoprogenitors during intramembranous bone formation; subsequent expression of *Sp7* is required for osteoblast precursor differentiation (Galea et al., 2021; Hojo and Ohba, 2022); and the phosphatase *Phospho1* is essential for inorganic phosphate incorporation and mineral deposition in the forming osteoid of differentiating osteoblasts (Dillon et al., 2019).

**Figure 6 –:**
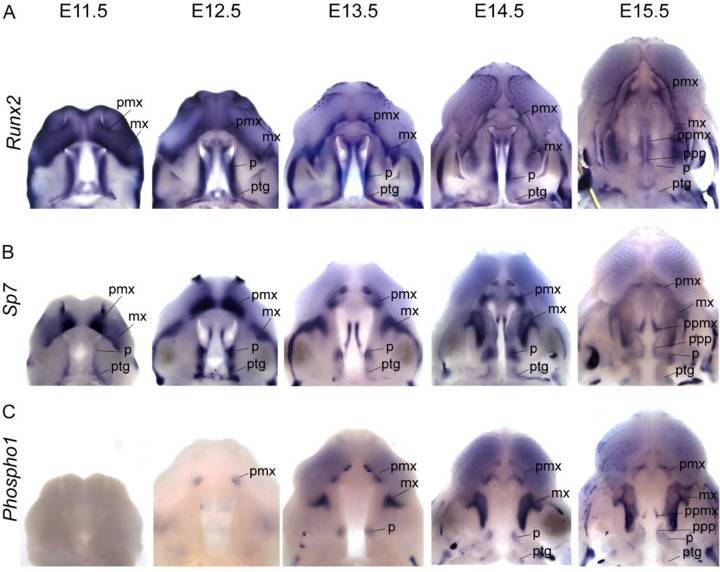
Segmental organization of skeletal specification and growth during A-P morphogenesis of the midface – WISH time course for (a) *Runx2*, (b) *Sp7*, and (c) *Phospho1* between E11.5 and E15.5. Expression of *Runx2* in osteoprogenitors highlights facial domains with osteogenic potential during midfacial morphogenesis. Expression of *Sp7* and *Phospho1* in committed and differentiating osteoblasts delineates the growth dynamics of individual skeletal anlagen during midfacial morphogenesis. Abbreviations: maxilla (mx), palatine (p), premaxilla (pmx), pterygoid (ptg), palatal process of the maxilla (ppmx), palatal process of the palatine (ppp)

Comparing the spatial expression dynamics for these marker genes showed that while *Runx2* signal does highlight some osteogenic domains, particularly in the premaxilla within the primary palate, it tended to be more broadly expressed within the early facial prominences. Within the developing secondary palate, expression of *Runx2* remained medial throughout this period, being restricted to domains adjacent to the molar tooth bud and medially along the adjoining palatal shelves (Fig. 6a).

The osteoblast-specific expression of both *Sp7* and *Phospho1* more precisely delineated the morphology of the emerging midfacial skeleton during this early period of midfacial outgrowth. At E11.5, *Sp7* was strongly expressed in bilateral domains within the mnp of the forming primary palate. Between E12.5 and E15.5, presaging premaxilla morphology, *Sp7* expression formed a cup-like region surrounding the developing incisors (Fig. 6b). At E11.5, *Sp7* expression in the MxP was observed in two domains. The anterio-lateral domain, interpreted as the presumptive maxilla, abutted the point of fusion with the mnp and extended laterally away from the oral cavity and choanae. The posterior domain spanned the length of the forming secondary palate, from the anterior limit abutting the choanae to the posterior-most palatal edge, and likely comprised the presumptive palatine and pterygoid bones. The antero-lateral domain of *Sp7* maintained proximity to the primary palate throughout the growth period, formed a new medial expansion (E12.5-E13.5) that extended posteriorly (E13.5-E15.5) within the growing anterior secondary palate (Fig. 6b). Between E12.5 and E15.5, anterior secondary palate elongation displaced the original posterior *Sp7* domain away from the primary palate. This posterior domain separated into two subdomains; the anterior subdomain remained associated with the posterior wall of the sinus cavity and gave rise to the palatine bone, while the posterior subdomain formed the pterygoid. Likely reflecting a temporal lag between initial osteoblast commitment and later differentiation, *Phospho1* was not detected in the midface at E11.5 and was observed only in the forming premaxilla at E12.5, followed by expression in progressively more posterior bone anlagen between E13.5 and E15.5 (Fig 6c).

These osteoblast-specific expression patterns indicated that the premaxilla, palatine, and pterygoid anlagen grew to acquire their characteristic adult morphology within the same domains where they were initially specified. However, the maxillary anlage was initially specified external to the oral cavity at the site of the future zygomatic plate. Only after anterior secondary palate elongation was initiated (E11.5–12.5) does a new portion of the maxillary anlage grow medially into the anterior secondary palate (E12.5–13.5) and extend posteriorly towards the palatine bone anlage (E13.5–15.5). The fact that *Phospho1* expression closely followed *Sp7* spatial dynamics within the maxillary anlage further supported the idea that *Sp7* expression patterns represent bone anlagen formation and expansion. Lastly, it is notable that the palatal processes of the maxilla and palatine bones formed within the medial region of the elevated palatal shelves, a domain that maintained high levels of *Runx2* expression during secondary palate growth (Fig. 6a), suggesting maintenance of a less differentiated osteoprogenitor population until palatal fusion was complete.

**Figure 7 –:**
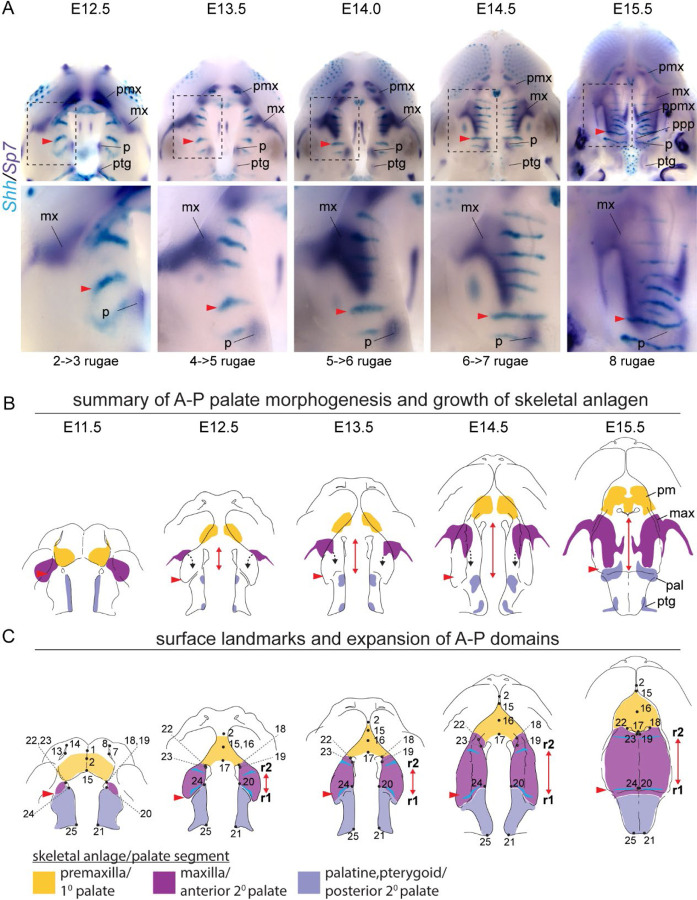
Double label RNA WISH time course for *Shh* (cyan) and *Sp7* (dark purple) between E12.5 and E15.5. (a) Expression of *Sp7* in committed and differentiating osteoblasts delineates the growth dynamics of individual skeletal anlagen and *Shh* expression in rugae provides a temporally ordered set of A-P landmarks (regions in dashed boxes enlarged below). (b) Summary model of skeletal growth dynamics during midfacial outgrowth. Growth of the premaxilla (yellow) and palatine (pale blue) anlagen towards their characteristic shape occurs largely at the site of initial specification. Following initial specification external to the oral cavity, the maxilla (purple) grows into the anterior of the anterior secondary palate (black dashed arrows) towards the position of ruga 1 (red arrowhead) and palatine as expansion of the anterior secondary palate (double headed red arrow) separates the primary and posterior secondary palate. (c) Summary of the position of epithelial landmarks (see also Fig. 2) selected to capture segmental growth dynamics of the primary palate (yellow) and anterior secondary palate (purple) and posterior secondary palate (pale blue) during midfacial outgrowth. Abbreviations: maxilla (mx), palatine (p), premaxilla (pmx), pterygoid (ptg), palatal process of the maxilla (ppmx), palatal process of the palatine (ppp)

Rugae morphogenesis provided a set of temporally ordered anatomical features that can be used to visualize A-P growth dynamics of the secondary palate. WISH double labeling of *Shh* and *Sp7* between E12.5 and E15.5 indicated the position of specific rugae relative to developing bone anlagen (Fig. 7a). These data further demonstrated that the initial site of maxillary specification (i.e. the future zygomatic plate) maintained a position external to the oral cavity and adjacent to the anterior most rugae during secondary palate elongation (Fig. 7a). Notably, medial maxillary anlage expansion into the secondary palate occurred around E13.5, after initial A-P growth of the anterior secondary palate and formation of 3–4 rugae anterior to ruga 1, and thus uniquely coupled to RGZ growth dynamics. The maxillary anlage grew posteriorly towards ruga 1 and the palatine anlage at the same time as subsequent rugae formation (E13.5-E14.5; Fig. 7a).

In contrast, ruga 1 and the palatine anlage initially formed at the E11.5 anterior extent of the secondary palate and maintained their proximity to each other throughout palatal development even as they were displaced posteriorly between E11.5 and E15.5 (Fig. 7a). The palatine and pterygoid bones later formed within the portion of the secondary palate that was already present at the onset of secondary palate morphogenesis. These results indicated that the posterior portion of the secondary palate and its associated anlage were present at the E11.5 onset of secondary palate morphogenesis, while the anterior secondary palate and associated anlage did not contribute substantially to the secondary palate until later (Fig. 7b). The combination of these morphogenetic results indicated that the meeting of the maxilla and palatine anlagen was achieved predominantly by posterior growth of the maxilla towards the palatine, which was itself being displaced posteriorly by anterior secondary palate elongation. By E15.5, the maxilla and palatine anlagen met to form the maxillary-palatine suture subjacent to ruga 1 (see also Supporting Fig. 1).

Our results collectively revealed that the segmental relationship between three palatal regions and associated bone anlagen was already present at the onset of palatal morphogenesis. Additionally, these results highlighted the previously unappreciated correlation of maxillary growth to rugae formation as well as the greater proportional expansion of the anterior secondary palate between E11.5 and E15.5 (summarized in Fig 7C). Based upon the spatial growth and differential A-P dynamics revealed by our marker analysis, we defined a set of surface landmarks intended to quantify the baseline characteristics and segmental differences in A-P tissue domains underlying midfacial growth, within the context of overall midfacial elongation.

### Interstrain Comparisons of Facial and Palatal Growth

**Figure 8 –:**
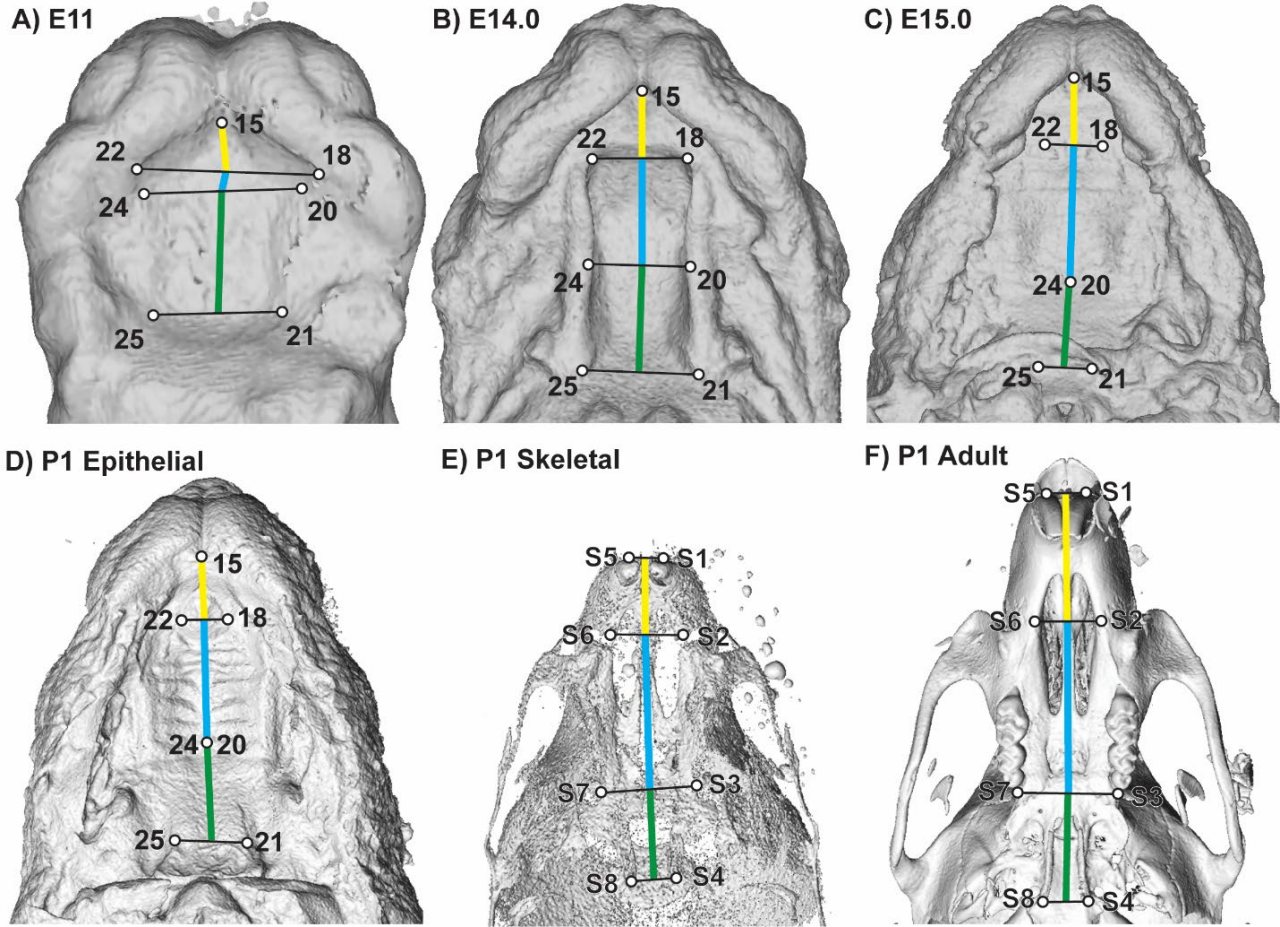
Palate segment length measurements – Identification of landmarks used to estimate midline lengths of the primary palate (yellow), anterior secondary palate (blue), and posterior secondary palate (green) on (a) E11.0, (b) E14.0, (c) E15.0, (d) P1 epithelial palatal surfaces, (e) P1 skeletal surfaces, and (f) adult skeletal surfaces. See also Supporting Tables 1 and 2.

Based on our findings that each upper jaw bone developed within one of the three palatal segments and these segments contributed somewhat independently to midfacial A-P elongation, we hypothesized that early differences in the proportional growth of the three palatal segments produces strain specific differences in the contributions of specific bones to upper jaw length. If true, previously identified differences in adult upper jaw morphology should be present by the end of the critical embryonic A-P elongation period (i.e., by E15.5). We completed a morphometric comparison of C57, NOD, and PWK strain embryonic midfacial shapes and a comparison of proportional palatal segment lengths across embryonic and postnatal stages (Fig. 8) based on the direct relationship between segmental borders identified in the epithelial surfaces and the specific bones that develop within the palatal segments.

Since there is known variation in the speed of development between mouse strains and within litters, we standardized our cross-strain ontogenetic analysis by embryonic developmental age rather than gestational age. Limb bud outline-based developmental ages correlated strongly with the number of days after plug identification for each measured strain. Though the correlation coefficients for C57 mice (0.98), NOD (0.93) and PWK (0.90) were high, there was a larger difference between limb age estimates and copulatory plug-based estimates of embryonic age for our PWK samples. The C57 specimen limb-based developmental ages were an average of 0.26 days younger than our copulatory plug estimates, while NOD and PWK limb ages were an average of 0.30 days and 1.09 days younger, respectively. The major divergence for PWK was likely driven by a slower speed of embryonic development in this wild-derived strain compared to the two common lab strains (Murray et al., 2010). Even so, standardization by developmental age should allow for improved direct comparisons between all strains.

**Figure 9 –:**
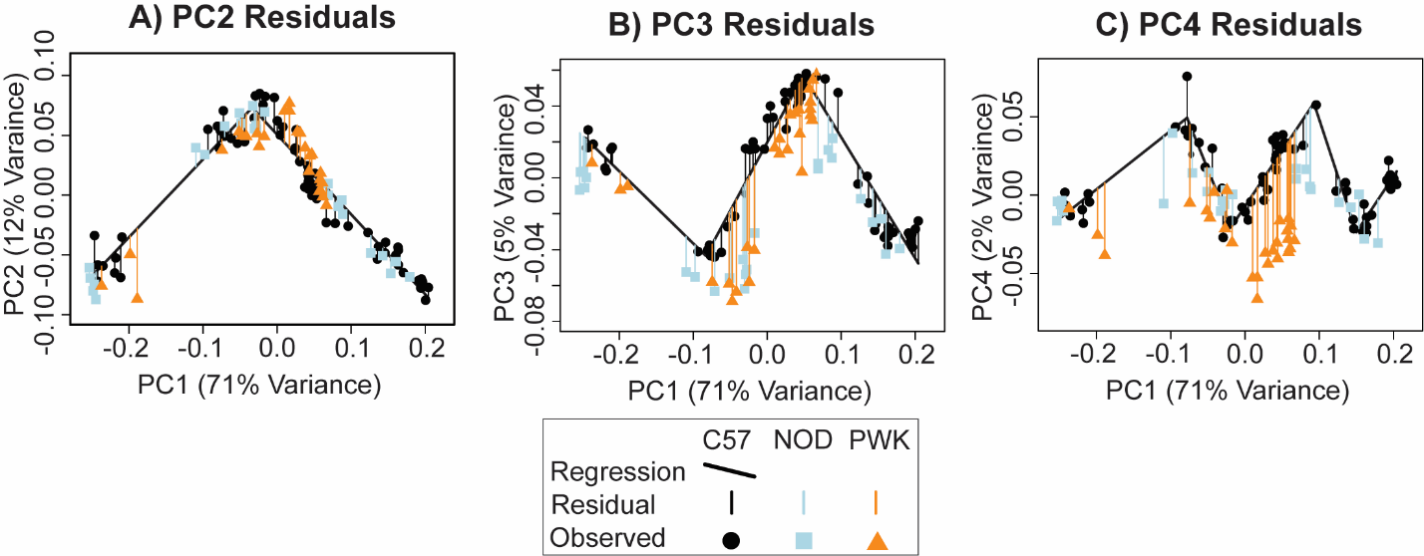
Specimen PC score residuals – The principal component (PC) score residuals for each specimen, relative to the scores predicted from C57 strain specific segmented linear regressions of (a) PC2, (b) PC3, and (c) PC4 on the specimen’s PC1 score. In this way, the C57 facial shape growth trajectory serves as a baseline with which to compare the shape of NOD and PWK specimens, based on the fact that the PC1 axis is strongly associated with facial shape ontogeny from developmental age E11 to E15.

We compared the midfacial shape of NOD and PWK specimens with C57 specimens along the principal axes of facial shape variation. This comparison indicated that mice with similar limb-based developmental age estimates tended to fall near each other along the first and second principal components, regardless of strain. As these axes represented 82% of facial shape variance across our sample, all mouse strains displayed major similarities in ontogenetic shape change across this period (Supporting Fig. 2A). However, across ontogenetic time (as represented by PC1 score) NOD and PWK specimens had significantly lower PC3 values compared to the C57 baseline (Fig. 9b). PWK specimens also had significantly lower PC4 values (Fig. 9c).

**Figure 10 –:**
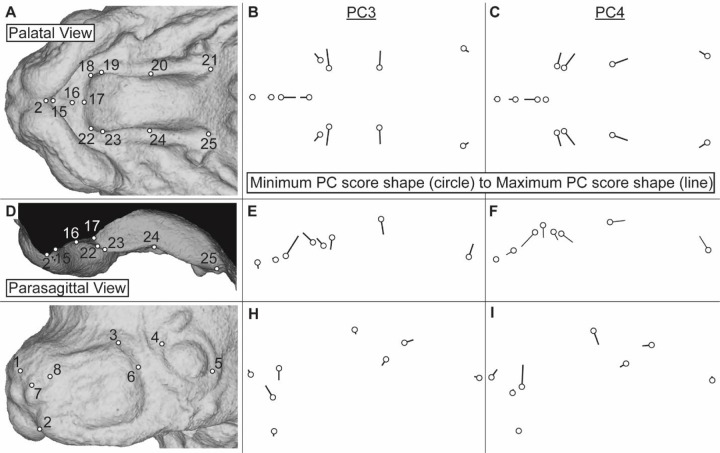
Facial shape variation associated with PC3 and PC4 – Representation of the pattern of shape variation associated with the third major axis and fourth major axis of embryonic facial/palatal shape variation, from (a-c) palatal, (d-f) parasagittal, and (g-i) facial views. Superficial tissue landmarks are identified on an example E14.5 specimen to assist with interpretation of the landmark vectors that represent differences in minimum (circle) and maximum (end of line) PC scores. See Supporting Fig. 2 for a plot of embryonic specimens along these major axes of embryonic shape variation.

We visualized how these identified significant differences in PC3 or PC4 scores were reflected within facial and palatal morphology by comparing shapes represented at the minimum and maximum end of these major shape axes (Fig. 10). A minimum PC3 score was associated with the two nostril landmarks (LM7 & 8) being relatively distant in the superior/inferior and lateral directions. Lower PC3 score was also associated with relative proximity of the posterior whisker margin (LM6) to the anterior canthus (LM4) (Fig. 10h). Within the palate, lower PC3 scores were associated with a relatively medial anterior secondary palatal shelf, suggesting more medial palatal outgrowth (proportional to overall palatal length) at a given developmental stage (Fig. 10b). The superior-inferior position of the palatal landmarks indicated that the secondary palate was more highly angled (less flat) for specimens with low PC3 scores (Fig. 10e). Because of their generally lower PC3 scores, we anticipated more vertical nostrils in NOD and PWK mice with more highly angled palatal shelves in greater medial proximity to each other, when compared to C57 mice within a given developmental age.

A minimum PC4 score was associated with the whole nasal region (LM1, LM8) being positioned more inferiorly and the posterior edge of the whisker region (LM3, LM6) more superiorly when compared to the other facial and palatal features (Fig. 10i, Supporting Fig. 4). Within the palate, a low PC4 score was associated with a relatively anterior location of ruga 1 between anterior and posterior secondary palate (LM20 & 24) and a relatively posterior position of the posterior edge of the posterior secondary palate (LM21 & 25 within Fig. 10c). Low PC4 scores were also associated with more medially positioned anterior secondary palate landmarks that are closer to the boundary with the primary palate. Because of their generally lower PC4 scores, this suggested that PWK embryos display a relatively low superior nasal region, a medially expanded anterior secondary palatal shelf with a relatively short anterior secondary palate and relatively long posterior secondary palate, when comparisons are made within a given developmental age.

**Figure 11 –:**
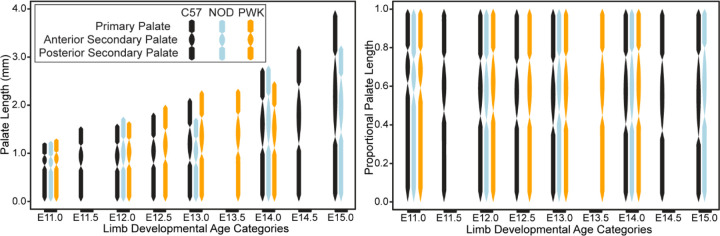
Embryonic palate segment length across ontogeny – The midline projected strain-specific mean lengths of the primary palate, anterior secondary palate, and posterior secondary palate, presented for each limb-based developmental stage category (E11-E15). Raw length values (in millimeters) are presented on the left, while palatal segment length relative to overall midline palate length are presented on the right.

**Figure 12 -:**
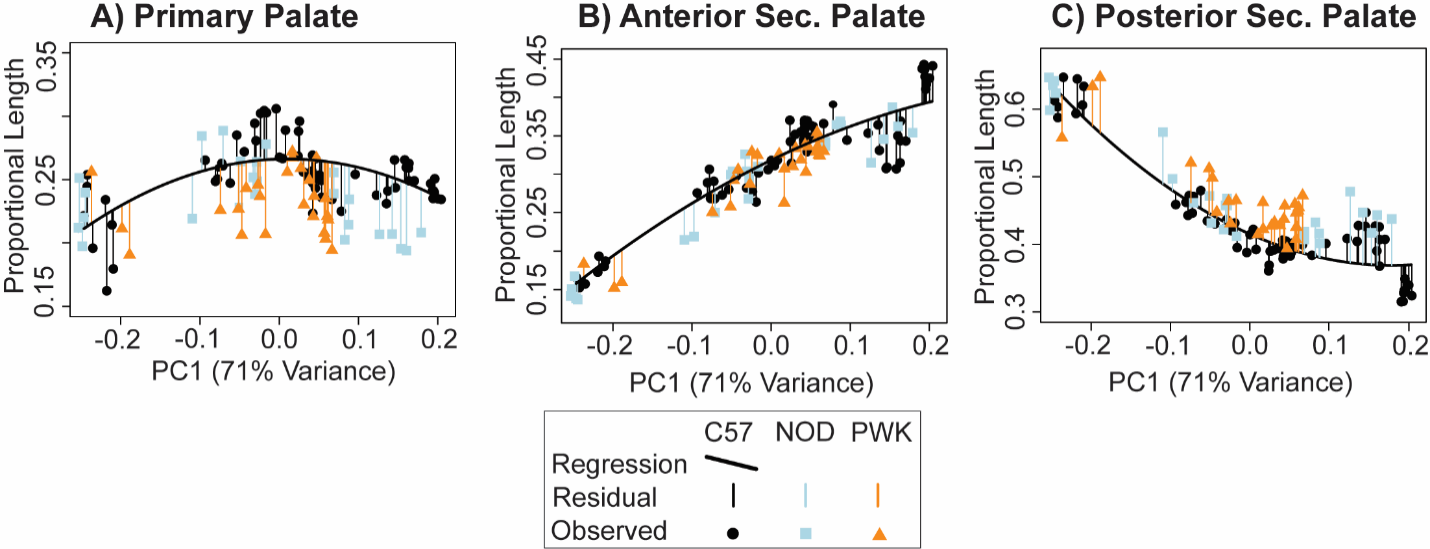
Specimen palate segment length residuals – The proportional contributions of each palate length segment (shapes), as compared to the proportional contributions predicted from C57 strain specific linear regressions (curved lines) of proportional (a) primary palate, (b) anterior secondary palate, and (c) posterior secondary palate midline lengths versus the specimen’s PC1 score. Vertical lines represent the residual of each specimen from C57 regression. In this way, the C57 palatal segment growth trajectory serves as a baseline that NOD and PWK specimens were compared to, based on the fact that the PC1 axis is strongly associated with facial shape ontogeny from E11 to E15.

A comparison of embryonic palatal segment lengths (Fig. 8a-c) across C57, NOD and PWK samples indicated that all three mouse strains display parallel ontogenetic changes 1) in overall palatal length and 2) in the proportional contributions of palatal regions to overall palatal length during the period of early palatal morphogenesis (Fig. 11). However, comparisons of regression residuals of proportional segment length versus facial shape PC1 score (as a proxy for developmental age) indicated small but significant differences in palatal segment proportions between mouse strains. The NOD and PWK primary palate proportional length values were significantly lower than for C57 (p-values: 0.012 (NOD vs C57), <0.001 (PWK vs C57)) (Fig. 12a), while they also had significantly higher posterior secondary palate values than C57 (p-values <0.001) (Fig. 12c). This indicated that the primary palate contributed relatively less and the posterior secondary palate contributed proportionally more to total embryonic palate length in these two mouse strains, although visual assessment suggested these differences do not occur at the earliest measured developmental ages (Fig. 11b). Significantly lower anterior secondary palate values for NOD and PWK (p-values: 0.029 (NOD vs C57), 0.023 (PWK vs C57) indicated that the anterior secondary palate represented a lower proportion of the total palate length within these strains (Fig. 12b), although these differences seemed less pronounced than those noted for the other two segments (Fig. 11b). Taken together, these general trends suggested that C57 mice had proportionally longer primary palates and proportionally shorter posterior secondary palates than NOD and PWK mice, with a trend towards longer anterior secondary palates by the time of medial palatal shelf fusion (Fig. 12).

### Interstrain Comparisons of Postnatal Palatal Segments

**Figure 13 –:**
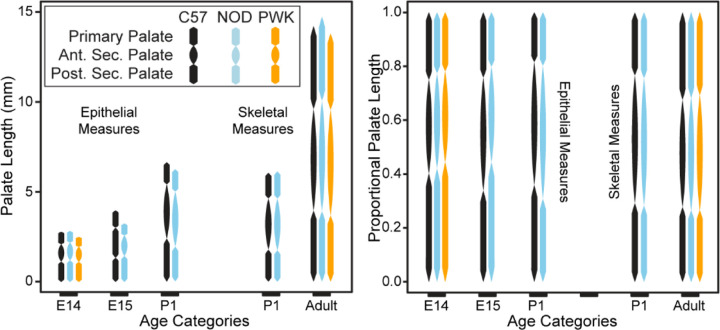
Interstrain comparisons of palate segment lengths in older specimen – The midline projected strain-specific mean lengths of the primary palate, anterior secondary palate, and posterior secondary palate, based on epithelial landmark measures for limb based developmental stages E14 and E15, as well as for P1 specimens. Analogous length measures of primary palate derived premaxilla, anterior secondary palate derived maxilla, and posterior secondary palate derived palatine/pterygoid bones are presented for P1 and adult specimens. Raw length values (in millimeters) are presented on the left, while palatal segment length relative to overall midline palate length are presented on the right.

We compared C57 and NOD proportional palatal segment lengths at P1 based on epithelial surface landmarks and skeletal landmarks of associated bony elements. Based on the P1 epithelial surface landmarks (Fig. 8d), C57 had a proportionally shorter anterior secondary palate and a proportionally longer posterior secondary palate (p-values <0.01) than NOD (Fig. 13; Table 2). This differed from the embryonic pattern where C57 specimens generally displayed proportionally shorter posterior secondary palates than NOD specimens. The significant differences in epithelially-measured proportional segment contributions between NOD and C57 mice at P1 appeared largely based on differences in the measured length (in mm) of the posterior secondary palate (Fig. 13).

A comparison of proportional palatal bone lengths in P1 samples (Fig. 8e) indicated that C57 has a proportionally shorter premaxilla (associated with primary palate) and proportionally longer palatine/pterygoid bones (associated with posterior secondary palate) (p-values <0.01) than NOD (Fig. 13; Table 2). A similar comparison of proportional palatal bone lengths (Fig. 8f) in 8–12 week old adult specimens of all three strains revealed that the premaxillary (primary palate) contribution to total palate length is significantly lower in PWK (p-value: < 0.001) and NOD (p-value: 0.044) compared to C57, with the opposite true for the maxillary (anterior secondary palate) portion of the palate (p-values: < 0.001) (Fig. 13). The palatine/pterygoid (posterior secondary palate) portion was proportionally shorter in PWK and NOD when compared to C57 (p-values: < 0.001) (Fig. 13). The PWK average proportional measures of premaxilla and maxilla showed a more extreme divergence from C57 than in the NOD mouse comparison.

Overall, there were some ontogenetic changes related to strain differences for proportional palatal segment lengths from the embryonic period to P1 and then again between P1 and adulthood. NOD and C57 showed parallel postnatal changes in palatal bone contributions across the postnatal period, where the posterior secondary palate contribution remained fairly stable (~27–29%), but the primary palate contribution increased from ~24% to ~32% and the anterior secondary palate contribution decreased from ~47% to ~40–42% (Fig. 13; Table 2). During the postnatal period, the entire mouse and its palate grew substantially, but premaxilla growth contributed more than the maxilla growth to overall postnatal palatal elongation. Although the A-P growth of the anterior secondary palate was a critical driver for midfacial growth between E11.5 and E15.5, outgrowth of the premaxilla may have played a larger role in postnatal midfacial outgrowth.

## Discussion

We provided here a multifaceted illustration of normal murine midfacial growth dynamics within the secondary palate and upper jaw bones during the earliest stages of palatal growth, followed by subsequent changes in upper jaw bone proportions in newborn and adult mice. By tracking the position of the RGZ, each palatal ruga, and palatal bone precursor populations across the earliest phases of palatal A-P elongation, our results confirmed that the first-formed palatal ruga, which sits at an important border of regulatory gene expression, also represents a consistent morphological boundary between the presumptive maxilla of the anterior secondary palate and the presumptive palatine and pterygoid bones of the posterior secondary palate. The process of rugae morphogenesis is coupled to maxillary osteogenesis during anterior secondary palate expansion. Also, from the time of its formation, ruga 1 represents the future position of the maxillary-palatine suture.

We tested the hypothesis that proportional differences in the earliest embryonic phases of palatal segment A-P elongation underlie strain-specific differences in the adult contributions of the premaxilla, maxilla, and palatine/pterygoid bones to upper jaw length. While these early morphogenetic processes are critical for normal midfacial outgrowth, our ontogenetic comparison of epithelial and skeletal palatal anatomy across three mouse strains indicated that adult-like strain-specific differences in upper jaw morphology are not present before or at birth. However, the significant strain-specific differences that were identified during embryonic palatal morphogenesis are a useful foundation for understanding the impact of background genetic effects on facial shape and elongation.

### Normal Upper Jaw and Palatal Elongation

Between mouse E11.5 and E15.5, as overall head size increases, the fused facial prominences grow dramatically outward to form an elongated snout. It is well known that A-P elongation of the palate is a necessary component of the integrated processes of midfacial outgrowth. However, we previously lacked a detailed picture of the earliest spatiotemporal dynamics of intramembranous skeletal specification and differentiation in the mammalian midface. Our combined morphometric and whole mount gene expression analysis spanned the period of secondary palate morphogenesis, providing a detailed description of early intramembranous skeletal specification and differentiation within the context of the surrounding palatal segments and rugae formation. This work indicated that the relationship between three longitudinal palate segments and associated upper jaw bones was already established during the earliest phases of palatal morphogenesis. These results also illustrated how differential growth of the palatal segments contribute to overall palate length in mice and likely in other mammals. While all three palatal segments elongated between E11.5 and E15.5, the proportional elongation of the anterior secondary palate was notably more pronounced than that of the primary or posterior secondary palate and was accompanied by rugae formation at the RGZ and elongation of the maxillary bone primordium.

Our results verified that ruga 1 forms at an important gene expression boundary between the anterior and posterior secondary palate. They also clarified previous hypotheses regarding the anatomical relationship between the position of ruga 1, the hard verus soft palate, and the maxillary-palatine suture (Pantalacci et al., 2008). Our results established that throughout palate morphogenesis, ruga 1 represents a stable morphological boundary positioned at the future maxillary-palatine suture and not the more posterior soft palate. Posteriorly, ruga 1 maintained a position at the anterior edge of the palatine anlage as both are posteriorly displaced between E11.5 and E15.5. This implied that at E11.5 the secondary palate was initially comprised of only the posterior secondary palate domains that will give rise to the palatine and pterygoid bones. Anterior to ruga 1, growth of the maxillary anlage into the secondary palate only occurred as anterior secondary palate expansion displaced the original site of maxillary specification anteriorly away from ruga 1. This suggested that cellular cues in the proximity of ruga 1 may initially inhibit maxillary bone formation. Expansion of the palatal portion of the maxillary anlage appeared uniquely coupled to dynamics within the RGZ of anterior secondary palate elongation, meaning this critical period of RGZ associated morphogenesis likely plays a major role in determining the proportional contribution of the maxilla to the upper jaw within a broad evolutionary context.

Differences in the formation and anatomy of two major upper jaw sutures may also be related to segmental differences in early bone growth. The premaxilla and maxilla were specified in adjacent tissue domains and maintained close apposition throughout palate morphogenesis, suggesting equal contributions to the formation of the premaxillary-maxillary suture. Conversely, the palatal portion of the maxilla grew posteriorly towards the palatine anlage at rugae 1, suggesting that maxillary growth (and likely RGZ dynamics) played a larger role than palatine growth in determining maxillary-palatine suture position. Different growth dynamics at these sutures may lead to a more vertical premaxillary-maxillary suture versus a more oblique maxillary-palatine suture with substantial A-P overlap of the maxilla and palatine bones. Suture formation also provides a critical niche for facial skeletal progenitors (Zhao et al., 2015). Thus, it is possible that early differences in segmental contributions to palate and upper jaw sutures may also contribute to differences in postnatal dynamics of midfacial growth and remodeling (Enlow and Bang, 1965; Kurihara et al., 1980; Sarnat, 1997; Martinez-Maza et al., 2013; Vora et al., 2015; Maga, 2016). Further support for this hypothesis comes from recent work that showed the widespread presence of Gli1+ mesenchymal stem cells and nascent bone formation (via calcein labeling) within the center of the adult maxillary-palatine suture and also in the maxillary bone, but a lack of stem cells and only sparse bone formation in the palatine bone (Luo et al., 2019). Given the established role of calvarial sutures in directing bone growth of the skull, further investigation of osteoprogenitor dynamics during premaxillary-maxillary and maxillary-palatine suture formation is warranted.

### Interstrain Palatal Growth Comparison

Our data illustrated 1) direct early associations between palatal segments and upper jaw bones morphogenesis and 2) that anterior secondary palate growth makes a central contribution towards total palatal elongation. This suggested a model where changes in embryonic A-P growth of the anterior secondary palate at the RGZ may produce the range of prognathism observed amongst mammals (Young et al., 2014). Given that A-P growth of the anterior secondary palate was associated with rugae formation, support for this mechanism is further strengthened by the fact that flat faced humans typically have 3–6 palatal rugae (Hauser et al., 1989; Jayasankar et al., 2016), mice have 8–9 rugae (Peterkova et al., 1987), and prognathic pigs have 20–25 rugae (Tonge and McCance, 1965). Based on this potential evo-devo relationship, we hypothesized that intra-species differences in the contribution of individual bones to upper jaw length would be produced by strain-specific differences in early embryonic A-P palatal segment elongation. Based on previously reported adult phenotypes (Percival et al., 2016), we hypothesized that C57 strain mice would have a relatively long primary palate and relatively short anterior secondary palate by E15, while NOD and PWK mice would display the opposite phenotype.

Although patterns of embryonic and postnatal palatal growth were broadly similar across our three inbred mouse strains (Figs. 11 & 13), we identified significant strain-associated differences in facial shape and palatal segment length. Embryonic facial shape differences (Figs. 4, 9, & 10; Table 2), as identified along major axes of variation, may represent more exaggerated versions of normal ontogenetic shifts in the relationship of various facial structures or shifts in the relative timing of normal developmental events occurring within different parts of the face. Significant strain-specific differences in palatal segments proportions were identified across the embryonic samples, with NOD and PWK mice having proportionally longer posterior secondary palates and shorter primary palates than C57 mice. This result broadly matched our hypothesis that embryonic C57 would have a relatively long primary palate, although without support for initially predicted differences in anterior secondary palate proportions. Therefore, these results demonstrated that there were significant embryonic differences in palatal proportions between the strains, but they do not coincide with the differences observed in adults.

The results also indicated that differences in proportional segment lengths between NOD and C57 did not remain consistent between the embryonic, neonatal (P1), and adult specimens. At P1, NOD had a relatively short posterior secondary palate (i.e. palatine and pterygoid) compared to C57, based on epithelial and skeletal measures. NOD also displayed a relatively long anterior secondary palate based on epithelial measures and relatively long premaxilla (i.e. primary palate) based on skeletal measures. This indicated a swap in proportional contributions for primary palate and posterior secondary palate between E15 and P1. Strain-specific premaxillary proportions flipped again between P1 and adult measurements.

Although changes within the RGZ growth dynamics of anterior secondary palate growth may be critical for major species-specific differences in prognathism, our results did not support the idea that this mechanism is responsible for the adult intraspecies differences in upper jaw morphology between our mouse strains. This may indicate that later prenatal (after E15) and postnatal growth processes play a major role in determining adult upper jaw bone proportions. Our results suggested that variation in postnatal growth of the primary palate derived premaxilla contributed more to postnatal elongation of the upper jaw than growth of the other palatal segments did. A larger sample of later embryonic ages (i.e., between E15.5 and P0) would allow us to determine when the initial NOD pattern of short primary and long posterior secondary palate changes to a pattern of long primary and short posterior secondary palate by P1.

### Concluding Statement

Our multifaceted illustration of normal midfacial growth dynamics confirmed a one-to-one relationship between palatal segments and upper jaw bones during the earliest stages of palatal growth, suggesting that the first formed ruga represents a consistent morphological boundary between anterior and posterior secondary palate bone precursors, being found at the future maxillary-palatine suture. Our results also indicated that interactions at the RGZ simultaneously drives rugae formation and elongation of the maxillary bone primordium within the anterior secondary palate, which more than doubles in length prior to palatal shelf fusion. Although it is likely that RGZ-driven A-P growth of the anterior secondary palate contributes to evolutionary changes in facial upper jaw morphology, this process was not solely responsible for the subtle strain-specific upper jaw bone proportions measured across three inbred mouse strains. However, significant strain-specific differences in early palatal segment elongation provide a useful foundation for understanding the impact of background genetic effects on facial morphogenesis. Our multifaceted analysis of ontogenetic trends in palatal and facial elongation provides a novel contextual framework and developmental perspective within which to evaluate the impact of both non-pathogenic and pathogenic genetic differences on midfacial growth and differentiation.

## Supplementary Material

Supplement 1

## Figures and Tables

**Table 1 –:** Sample size of µCT imaged and morphometrically analyzed embryo specimens by strain and forelimb based developmental age estimation, as estimated using eMOSS (Musy et al., 2018).

Limb-Based Developmental Age	C57	NOD	PWK
E11	8	12	4
E11.5	2	0	0
E12	7	3	6
E12.5	4	0	2
E13	9	3	6
E13.5	0	0	2
E14	26	6	6
E14.5	1	0	0
E15	9	4	0

**Table 2 –:** Average proportional palatal segment lengths for postnatal specimens, presented as percentages of total palatal length.

	C57			NOD			PWK
	P1 epithelial	P1 bone	Adult bone	P1 epithelial	P1 bone	Adult bone	Adult bone
Primary Palate / Premaxilla	18.4%	23.3%	32.4%	19.7%	24.4%	31.7%	30.7%
Ant. Sec. Palate / Maxilla	45.7%	47.5%	39.9%	49.5%	47.4%	41.8%	42.8%
Post. Sec. Palate / Palatine & Pterygoid	35.9%	29.2%	27.7%	30.8%	28.3%	26.5%	26.5%

## References

[R1] Adams D.C., Collyer M.L., Kaliontzopoulou A., 2020. geomorph: Software for geometric morphometric analyses. R package version 3.3.1. https://cran.r-project.org/package=geomorph

[R2] Boughner J.C., Wat S., Diewert V.M., Young N.M., Browder L.W., Hallgrímsson B., 2008. Short-faced mice and developmental interactions between the brain and the face. Journal of Anatomy 213, 646–662. 19094181 10.1111/j.1469-7580.2008.00999.x PMC2666134

[R3] Bush J.O., Jiang R., 2012. Palatogenesis: morphogenetic and molecular mechanisms of secondary palate development. Development 139, 231–243. 22186724 10.1242/dev.067082 PMC3243091

[R4] Cignoni P., Callieri M., Corsini M., Dellepiane M., Ganovelli F., Ranzuglia G., 2008. Meshlab: an open-source mesh processing tool., in: Eurographics Italian Chapter Conference. pp. 129–136. 10.2312/LocalChapterEvents/ItalChap/ItalianChapConf2008/129-136

[R5] Cobb S.N., O’Higgins P., 2004. Hominins do not share a common postnatal facial ontogenetic shape trajectory. Journal of Experimental Zoology Part B: Molecular and Developmental Evolution 302, 302–321. 15211688 10.1002/jez.b.21005

[R6] Collyer M.L., Adams D.C., 2018. RRPP: An R package for fitting linear models to high-dimensional data using residual randomization. Methods in Ecology and Evolution.

[R7] Dillon S., Staines K.A., Millán J.L., Farquharson C., 2019. How To Build a Bone: PHOSPHO1, Biomineralization, and Beyond. JBMR Plus 3, e10202. 10.1002/jbm4.10202 31372594 PMC6659447

[R8] Economou A.D., Ohazama A., Porntaveetus T., Sharpe P.T., Kondo S., Basson M.A., , 2012. Periodic stripe formation by a Turing mechanism operating at growth zones in the mammalian palate. Nat Genet 44, 348–351. 10.1038/ng.1090 22344222 PMC3303118

[R9] Enlow D.H., Bang S., 1965. Growth and remodeling of the human maxilla. Am J Orthod 51, 446–464. 10.1016/0002-9416(65)90242-3 14287831

[R10] Fedorov A., Beichel R., Kalpathy-Cramer J., Finet J., Fillion-Robin J.-C., Pujol S., , 2012. 3D Slicer as an image computing platform for the Quantitative Imaging Network. Magnetic Resonance Imaging 30, 1323–1341. 10.1016/j.mri.2012.05.001 22770690 PMC3466397

[R11] Fish J.L., 2019. Evolvability of the vertebrate craniofacial skeleton. Semin Cell Dev Biol 91, 13–22. 10.1016/j.semcdb.2017.12.004 29248471 PMC5999547

[R12] Galea G.L., Zein M.R., Allen S., Francis-West P., 2021. Making and shaping endochondral and intramembranous bones. Dev Dyn 250, 414–449. 10.1002/dvdy.278 33314394 PMC7986209

[R13] Geyer S.H., Mohun T.J., Weninger W.J., 2009. Visualizing vertebrate embryos with episcopic 3D imaging techniques. ScientificWorldJournal 9, 1423–1437. 10.1100/tsw.2009.154 20024516 PMC5823209

[R14] Hall J., Jheon A.H., Ealba E.L., Eames B.F., Butcher K.D., Mak S.-S., , 2014. Evolution of a developmental mechanism: Species-specific regulation of the cell cycle and the timing of events during craniofacial osteogenesis. Dev Biol 385, 380–395. 10.1016/j.ydbio.2013.11.011 24262986 PMC3953612

[R15] Hallgrímsson B., Jamniczky H., Young N.M., Rolian C., Parsons T.E., Boughner J.C., , 2009. Deciphering the Palimpsest: Studying the Relationship between Morphological Integration and Phenotypic Covariation. Evolutionary Biology 36, 355–376. 23293400 10.1007/s11692-009-9076-5 PMC3537827

[R16] Hammond N.L., Dixon M.J., 2022. Revisiting the embryogenesis of lip and palate development. Oral Dis 28, 1306–1326. 10.1111/odi.14174 35226783 PMC10234451

[R17] Hauser G., Daponte A., Roberts M., 1989. Palatal rugae. Journal of Anatomy 165, 237–249. 17103618 PMC1256673

[R18] Hilliard S.A., Yu L., Gu S., Zhang Z., Chen Y.P., 2005. Regional regulation of palatal growth and patterning along the anterior–posterior axis in mice. Journal of Anatomy 207, 655–667. 10.1111/j.1469-7580.2005.00474.x 16313398 PMC1571556

[R19] Hojo H., Ohba S., 2022. Sp7 Action in the Skeleton: Its Mode of Action, Functions, and Relevance to Skeletal Diseases. Int J Mol Sci 23, 5647. 10.3390/ijms23105647 35628456 PMC9143072

[R20] Jayasankar P., Banker A., Bhattacharya A., Gandhi R., Patel N., Parikh S., 2016. Quantitative and qualitative analysis of palatal rugae patterns in Gujarati population: A retrospective, cross-sectional study. J Forensic Dent Sci 8, 126. 10.4103/0975-1475.195110 28123265 PMC5210098

[R21] Kaucka M., Petersen J., Tesarova M., Szarowska B., Kastriti M.E., Xie M., , 2018. Signals from the brain and olfactory epithelium control shaping of the mammalian nasal capsule cartilage. Elife 7, e34465. 10.7554/eLife.34465 29897331 PMC6019068

[R22] Kawasaki M., Kawasaki K., Meguro F., Yamada A., Ishikawa R., Porntaveetus T., , 2018. Lrp4/Wise regulates palatal rugae development through Turing-type reaction-diffusion mechanisms. PLoS One 13, e0204126. 10.1371/journal.pone.0204126 30235284 PMC6147471

[R23] Kundrát M., Coria R.A., Manning T.W., Snitting D., Chiappe L.M., Nudds J., , 2020. Specialized Craniofacial Anatomy of a Titanosaurian Embryo from Argentina. Current Biology 30, 4263–4269.e2. 10.1016/j.cub.2020.07.091 32857974

[R24] Kurihara S., Enlow D.H., Rangel R.D., 1980. Remodeling reversals in anterior parts of the human mandible and maxilla. Angle Orthod 50, 98–106. 10.1043/0003-3219(1980)050&lt;0098:RRIAPO&gt;2.0.CO;2 6929173

[R25] Lee S.-H., Bédard O., Buchtová M., Fu K., Richman J.M., 2004. A new origin for the maxillary jaw. Developmental biology 276, 207–224. 15531375 10.1016/j.ydbio.2004.08.045

[R26] Leigh S.R., 2006. Cranial ontogeny of Papio baboons (Papio hamadryas). American Journal of Physical Anthropology 130, 71–84. 10.1002/ajpa.20319 16345071

[R27] Li Q., Ding J., 2007. Gene expression analysis reveals that formation of the mouse anterior secondary palate involves recruitment of cells from the posterior side. Int. J. Dev. Biol. 51, 167–172. 17294368 10.1387/ijdb.062212ql

[R28] Luo W., Yi Y., Jing D., Zhang S., Men Y., Ge W.-P., , 2019. Investigation of Postnatal Craniofacial Bone Development with Tissue Clearing-Based Three-Dimensional Imaging. Stem Cells and Development 28, 1310–1321. 10.1089/scd.2019.0104 31392933 PMC6767869

[R29] Maga A.M., 2016. Postnatal Development of the Craniofacial Skeleton in Male C57BL/6J Mice. J Am Assoc Lab Anim Sci 55, 131–136. 27025802 PMC4783629

[R30] Martinez-Maza C., Rosas A., Nieto-Díaz M., 2013. Postnatal changes in the growth dynamics of the human face revealed from bone modelling patterns. J Anat 223, 228–241. 10.1111/joa.12075 23819603 PMC3972044

[R31] Morris Z.S., Abzhanov A., 2021. Heading for higher ground: Developmental origins and evolutionary diversification of the amniote face. Curr Top Dev Biol 141, 241–277. 10.1016/bs.ctdb.2020.12.003 33602490

[R32] Murray S.A., Morgan J.L., Kane C., Sharma Y., Heffner C.S., Lake J., , 2010. Mouse Gestation Length Is Genetically Determined. PLoS ONE 5, e12418. 10.1371/journal.pone.0012418 20811634 PMC2928290

[R33] Musy M., Flaherty K., Raspopovic J., Robert-Moreno A., Richtsmeier J.T., Sharpe J., 2018. A quantitative method for staging mouse embryos based on limb morphometry. Development 145, 1–7. 10.1242/dev.154856 PMC5963863 29540505

[R34] Okano J., Suzuki S., Shiota K., 2006. Regional heterogeneity in the developing palate: morphological and molecular evidence for normal and abnormal palatogenesis. Congenital Anomalies 46, 49–54. 10.1111/j.1741-4520.2006.00103.x 16732762

[R35] Palmer A.R., Strobeck C., 1986. Fluctuating Asymmetry: Measurement, Analysis, Patterns. Annu. Rev. Ecol. Syst. 17, 391–421. 10.1146/annurev.es.17.110186.002135

[R36] Pantalacci S., Prochazka J., Martin A., Rothova M., Lambert A., Bernard L., , 2008. Patterning of palatal rugae through sequential addition reveals an anterior/posterior boundary in palatal development. BMC Developmental Biology 8, 116. 10.1186/1471-213X-8-116 19087265 PMC2637861

[R37] Pauws E., Hoshino A., Bentley L., Prajapati S., Keller C., Hammond P., , 2009. Tbx22null mice have a submucous cleft palate due to reduced palatal bone formation and also display ankyloglossia and choanal atresia phenotypes. Hum Mol Genet 18, 4171–4179. 10.1093/hmg/ddp368 19648291 PMC2758147

[R38] Percival C.J., Liberton D.K., Pardo-Manuel de Villena F., Spritz R., Marcucio R., Hallgrímsson B., 2016. Genetics of murine craniofacial morphology: diallel analysis of the eight founders of the Collaborative Cross. Journal of Anatomy 228, 96–112. 10.1111/joa.12382 26426826 PMC4694168

[R39] Peterkova R., Klepacek I., Peterka M., 1987. Prenatal development of rugae palatinae in mice: scanning electron microscopic and histologic studies. Journal of craniofacial genetics and developmental biology 7, 169–89. 3624420

[R40] Ponce de León M.S.P., Zollikofer C.P.E., 2001. Neanderthal cranial ontogeny and its implications for late hominid diversity. Nature 412, 534–538. 11484052 10.1038/35087573

[R41] R Core Team, 2021. R: A language and environment for statistical computing. R Foundation for Statistical Computing, Vienna, Austria [WWW Document]. URL https://www.R-project.org/

[R42] Sarnat B.G., 1997. Some methods of assessing postnatal craniofaciodental growth: a retrospective of personal research. Cleft Palate Craniofac J 34, 159–172. 10.1597/1545-1569_1997_034_0159_smoapc_2.3.co_2 9138513

[R43] Schneider R.A., 2015. Regulation of Jaw Length During Development, Disease, and Evolution. Curr Top Dev Biol 115, 271–298. 10.1016/bs.ctdb.2015.08.002 26589929

[R44] Selleri L., Rijli F.M., 2023. Shaping faces: genetic and epigenetic control of craniofacial morphogenesis. Nature Reviews Genetics 24, 610–626. 10.1038/s41576-023-00594-w 37095271

[R45] Singleton M., 2012. Postnatal Cranial Development in Papionin Primates: An Alternative Model for Hominin Evolutionary Development. Evolutionary Biology 39, 499–520. 10.1007/s11692-011-9153-4

[R46] Smith F.J., Percival C.J., Young N.M., Hu D., Schneider R.A., Marcucio R.S., , 2015. Divergence of craniofacial developmental trajectories among avian embryos. Developmental Dynamics. 10.1002/dvdy.24262 PMC4544654 25703037

[R47] Tonge C.H., McCance R.A., 1965. Severe undernutrition in growing and adult animals: 15. The mouth, jaws and teeth of pigs. British Journal of Nutrition 19, 361–372. 10.1079/BJN19650034 5891038

[R48] Vora S.R., Camci E.D., Cox T.C., 2015. Postnatal Ontogeny of the Cranial Base and Craniofacial Skeleton in Male C57BL/6J Mice: A Reference Standard for Quantitative Analysis. Front Physiol 6, 417. 10.3389/fphys.2015.00417 26793119 PMC4709510

[R49] Welsh I.C., Feiler M.E., Lipman D., Mormile I., Hansen K., Percival C.J., 2023. Palatal segment contributions to midfacial growth in three inbred mouse strains. 10.5061/dryad.ghx3ffbvb PMC12159334 39831750

[R50] Welsh I.C., Hagge-Greenberg A., O’Brien T.P., 2007. A dosage-dependent role for Spry2 in growth and patterning during palate development. Mechanisms of Development 124, 746–761. 10.1016/j.mod.2007.06.007 17693063 PMC2043129

[R51] Welsh I.C., Hart J., Brown J.M., Hansen K., Rocha Marques M., Aho R.J., , 2018. Pbx loss in cranial neural crest, unlike in epithelium, results in cleft palate only and a broader midface. Journal of Anatomy 233, 222–242. 10.1111/joa.12821 29797482 PMC6036936

[R52] Welsh I.C., O’Brien T.P., 2009. Signaling integration in the rugae growth zone directs sequential SHH signaling center formation during the rostral outgrowth of the palate. Developmental Biology 336, 53–67. 10.1016/j.ydbio.2009.09.028 19782673 PMC2789450

[R53] Wilson R., McGuire C., Mohun T., DMDD Project, 2016. Deciphering the mechanisms of developmental disorders: phenotype analysis of embryos from mutant mouse lines. Nucleic Acids Res 44, D855–861. 10.1093/nar/gkv1138 26519470 PMC4702824

[R54] Woronowicz K.C., Schneider R.A., 2019. Molecular and cellular mechanisms underlying the evolution of form and function in the amniote jaw. EvoDevo 10, 17. 10.1186/s13227-019-0131-8 31417668 PMC6691539

[R55] Young N.M., Hu D., Lainoff A.J., Smith F.J., Diaz R., Tucker A.S., , 2014. Embryonic bauplans and the developmental origins of facial diversity and constraint. Development 141, 1059–1063. 10.1242/dev.099994 24550113 PMC3929406

[R56] Yu K., Ornitz D.M., 2011. Histomorphological study of palatal shelf elevation during murine secondary palate formation. Developmental Dynamics 240, 1737–1744. 10.1002/dvdy.22670 21618642 PMC3275090

[R57] Yu L., Gu S., Alappat S., Song Y., Yan M., Zhang X., , 2005. Shox2-deficient mice exhibit a rare type of incomplete clefting of the secondary palate. Development 132, 4397–4406. 10.1242/dev.02013 16141225

[R58] Yuan Y., Loh Y.-H.E., Han X., Feng J., Ho T.-V., He J., , 2020. Spatiotemporal cellular movement and fate decisions during first pharyngeal arch morphogenesis. Sci Adv 6, eabb0119. 10.1126/sciadv.abb0119 33328221 PMC7744069

[R59] Zelditch M., Swiderski D., Sheets D.H., 2012. Geometric morphometrics for biologists: a primer, 2nd ed. Elsevier Academic Press, San Diego.

[R60] Zhao H., Feng J., Ho T.-V., Grimes W., Urata M., Chai Y., 2015. The suture provides a niche for mesenchymal stem cells of craniofacial bones. Nat Cell Biol 17, 386–396. 10.1038/ncb3139 25799059 PMC4380556

[R61] Zumpano M.P., Richtsmeier J.T., 2003. Growth-related shape changes in the fetal craniofacial complex of humans (Homo sapiens) and pigtailed macaques (Macaca nemestrina): a 3D-CT comparative analysis. American Journal of Physical Anthropology 120, 339–351. 12627529 10.1002/ajpa.10125

