# Palatal segment contributions to midfacial anterior-posterior growth

**DOI:** 10.1101/2023.10.03.560703

**Published:** 2024-06-28

**Authors:** Ian C. Welsh, Maria E. Feiler, Danika Lipman, Isabel Mormile, Karissa Hansen, Christopher J. Percival

**Affiliations:** Program in Craniofacial Biology, University of California at San Francisco, San Francisco, California 94143, USA; Department of Orofacial Sciences, University of California at San Francisco, San Francisco, California 94143, USA; Department of Anatomy, University of California at San Francisco, San Francisco, California 94143, USA; Interdepartmental Doctoral Program in Anthropological Sciences, Stony Brook University, Stony Brook, NY 11790; Department of Cell Biology and Anatomy, University of Calgary; Interdepartmental Doctoral Program in Anthropological Sciences, Stony Brook University, Stony Brook, NY 11790; Program in Craniofacial Biology, University of California San Francisco, San Francisco, CA 94143; Department of Orofacial Sciences, University of California San Francisco, San Francisco, CA 94143; Department of Anatomy, University of California San Francisco, San Francisco, CA 94143; Department of Anthropology, Stony Brook University, Stony Brook NY 11794

**Keywords:** palatogenesis, secondary palate, rugae, face elongation, *Mus musculus*, RRID:IMSR_JAX:003715, RRID:IMSR_JAX:001976, RRID:IMSR_JAX:000664

## Abstract

Anterior-posterior (A-P) elongation of the palate is a critical aspect of integrated midfacial morphogenesis. Reciprocal epithelial-mesenchymal interactions drive secondary palate elongation that is coupled to the periodic formation of signaling centers within the rugae growth zone (RGZ). However, the relationship between RGZ driven morphogenetic processes, the differentiative dynamics of underlying palatal bone mesenchymal precursors, and the segmental organization of the upper jaw has remained enigmatic. A detailed ontogenetic study of these relationships is important, because palatal segment growth is a critical aspect of normal midfacial growth, can be modified to produce dysmorphology, and is a likely basis for evolutionary differences in upper jaw morphology. Variation in palatal-segment specific growth may also underlie known differences in palatal segment proportions between inbred mouse strains. We completed a combined whole mount gene expression and morphometric analysis of normal murine palatal growth dynamics and their association with palatal segment elongation and resulting upper jaw morphology. Our results demonstrated that the first formed palatal ruga (ruga 1), found just posterior to the RGZ, maintained an association with important nasal, neurovascular and palatal structures throughout early midfacial development; suggesting that these features are positioned at a proximal source of embryonic midfacial directional growth. Our detailed characterization of midfacial morphogenesis revealed a one-to-one relationship between palatal segments and upper jaw bones during the earliest stages of palatal elongation. Growth of the maxillary anlage within the anterior secondary palate is uniquely coupled to RGZ-driven morphogenesis that more than doubles the length of this palatal segment prior to palatal shelf fusion. Our results also demonstrate that the future maxillary-palatine suture, approximated by the position ruga 1 and consistently associated with the palatine anlage, forms predominantly via the posterior differentiation of the maxilla within the expanding anterior secondary palate. Our complementary ontogenetic comparison of three inbred mouse strains identified small but significant strain-specific differences in early embryonic palatal segment contributions to the upper jaw. Although early palatal segment specific growth is not primarily responsible for adult differences in upper jaw morphology between these strains, our ontogenetic series of measurements provide a useful foundation for understanding the impact of background genetic effects on facial shape and elongation. In combination, our results provide a novel and particularly detailed picture of the earliest spatiotemporal dynamics of intramembranous midfacial skeletal specification and differentiation within the context of the surrounding palatal segment A-P elongation and associated rugae formation.

## Introduction

**Figure 1 –:**
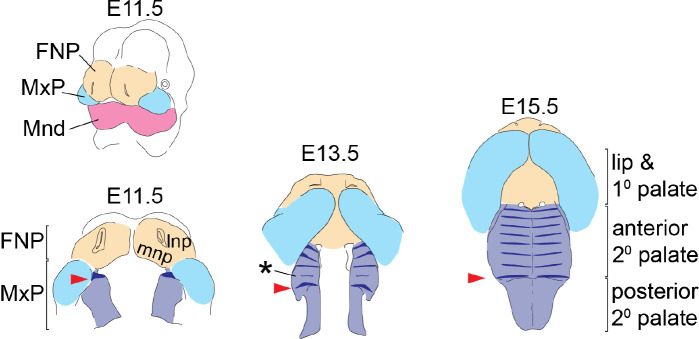
Tissue origins of the midfacial complex and rugae position during secondary palate morphogenesis – The upper and lower jaws are formed from the frontonasal process (FNP - tan), and branchial arch 1 derived maxillary and mandibular processes (MxP - pale blue and Mnd – magenta, respectively). From E11.5 to E15.5, outgrowth and fusion of the medial nasal process (mnp) with the superficial portion of the MxP frame out the lip and primary (1°) palate, while the A-P elongation and medially directed growth of the palatal shelves from the internal portion of the MxP gives rise to the secondary palate (2° pal – light purple). *Shh* expression (dark blue) highlights the dynamics of rugae formation and illustrates regional expansion of the anterior secondary palate. At E11.5, ruga 1 (red arrowheads) forms at the anterior extent of the nascent palatal shelf and subsequently defines the caudal end of the rugae growth zone (asterisk) where new rugae form prior to being displaced anteriorly. Additional abbreviation: lateral nasal process (lnp).

Morphological variation of the midfacial complex, which consists of the nose, upper jaw, cheek, and palate, is a defining aspect of both intra- and inter-specific differences in facial shape. Basic facial shape is the result of cranial neural crest (CNC)-derived facial prominence outgrowth and fusion, which occur between embryonic day (E) 10 and E15 in mice (Fig. 1). Initial fusion between the medial nasal processes (mnp) of the frontonasal process (FNP) and the anterior maxillary processes (MxP) gives rise to the primary palate and lip, producing a unified upper jaw from previously separated tissues. Secondary palate morphogenesis then begins through outgrowth of the nascent palatal shelf along the medial aspect of the MxP. Significant palatal growth along the anterior-posterior (A-P) axis is accompanied by vertical growth of the palatal shelves prior to their elevation and medial fusion dorsal to the tongue, after which the palatal shelf separates the oral and nasal cavities (Bush and Jiang, 2012; Hammond and Dixon, 2022). Disproportional growth of FNP or MxP prior to fusion can result in cleft lip and palate (Young et al., 2014), while poor coordination between the multiple growth axes of MxP-derived tissues can prevent secondary palate closure even when the palatal shelves maintain competency to fuse (Kouskoura, et al., 2013).

Species-specific differences in the size, shape, and growth trajectories of facial prominences are identifiable during the earliest phases of facial morphogenesis, prior to skeletal differentiation, often presaging interspecies differences in adult facial shape (Selleri and Rijli, 2023). After initial facial prominence fusion, differential A-P growth of FNP and MxP derived tissues is a major basis for evolutionary differences in midfacial prognathism (Young et al., 2014); possibly based on the balance of CNC progenitor self-renewal versus osteoblast differentiation (Hall et al., 2014; Morris and Abzhanov, 2021; Schneider, 2015). The regional basis for facial elongation varies across taxa; primarily driven by MxP growth in mammals and by FNP growth in birds (Young et al., 2014), indicating different palatal segment contributions.

The three longitudinal segments of the palate are called the primary palate, anterior secondary palate, and posterior secondary palate (Fig. 1). The boundary between primary palate and anterior secondary palate is the posterior edge of the fused mnp. Although A-P organization of the secondary palate can be defined by the presence of boney versus muscular tissue (i.e. the hard versus soft palate) or modes of palatal shelf elevation and closure (reviewed by Yu and Ornitz, 2011), we define secondary palate segments by molecular differences in tissue patterning and cell signaling competence (Hilliard et al., 2005; Hammond and Dixon, 2022).

The A-P expression domains of certain transcription factors (TFs) and intercellular signaling molecules within CNC-derived mesenchyme are important for A-P organization and growth during palatal morphogenesis. For example, *Msx1* and *Shox2* are exclusively expressed in the anterior secondary palate while *Barx1* and *Tbx22* are exclusive to the posterior secondary palate within this mesenchyme (Hilliard et al., 2005; Okano et al., 2006; Welsh et al., 2018). Mutations of *Shox2* or *Tbx22* have resulted in midline clefts of the anterior or posterior palate, respectively (Yu et al., 2005; Pauws et al., 2009), although regional expression is maintained in genetically engineered mice with severe segmental growth defects (Welsh et al., 2018). The A-P expression boundary of these and other factors is found at the first formed palatal ruga (Hilliard et al., 2005; Li and Ding, 2007; Welsh et al., 2007; Welsh and O’Brien, 2009; Welsh et al., 2018; Hammond and Dixon, 2022), one of multiple parallel epithelial thickenings on the anterior secondary palate.

This first ruga (ruga 1 numbered according to Welsh et al., 2007, Welsh and O’Brien, 2009; although Pantalacci et al., 2008 use a different numbering system) forms at E11.5 on the anterior extent of the nascent secondary palate (red arrow in Fig. 1). Remaining rugae form sequentially within the rugae growth zone (RGZ) located just anterior to ruga 1 (Welsh and O’Brien, 2009), via a Turing type mechanism (Economou et al., 2012; Kawasaki et al., 2018). Rugae are centers of Sonic Hedgehog signaling (SHH), which is critical for anterior secondary palatal patterning and A-P growth. As the secondary palate completes midline fusion at E15.5, the last formed ruga appears adjacent to ruga 1 in the middle of the secondary palate while each precedingly formed ruga is found at an increasingly anterior position along the anterior secondary palate (Welsh and O’Brien, 2009).

Based upon the dynamics of rugae formation, palate growth, and patterning gene expression, Pantalacci (et al., 2008) hypothesized that ruga 1 represents a distinct morphological boundary related to either the future hard versus soft palate boundary or the maxillary-palatine suture. However, the degree to which rugae formation and regional A-P patterns of secondary palate gene expression are coordinated with palate skeletal differentiation remain unclear. To address this knowledge gap, we mapped markers associated with the specification and differentiation of osteoblasts within the context of secondary palate segmental organization, across a developmental time course spanning mouse secondary palate morphogenesis and upper jaw outgrowth (E11.5-E15.5). Our results indicate that ruga 1 is coincident with the future position of the maxillary-palatine suture, illustrate the one-to-one palatal segment origins of upper jaw bones, and reveal a previously unappreciated coupling of maxillary formation to rugae morphogenesis during elongation of the anterior secondary palate.

Given 1) the strong early one-to-one association between forming upper jaw bones and palatal segment specific growth factor expression and 2) that embryonic morphogenesis is critical for the production of typical facial shape (e.g., Hilliard et al., 2005; Kaucka et al., 2018; Yuan et al., 2020), it’s possible that early differences in the longitudinal growth of the three palatal segments underlie inter- and intraspecies differences in upper jaw morphology. Specifically, we hypothesized that genetically based differences in palate segment-specific A-P outgrowth would explain previously identified variation in the contribution of the premaxilla, maxilla, and palatine bones to adult upper jaw morphology of inbred mouse strains.

C57BL/6J (RRID:IMSR_JAX:000664) inbred strain adults have a relatively long premaxilla, while NOD/ShiLtJ (RRID:IMSR_JAX:001976) have a relatively long maxilla, making them a straightforward comparison. The PWK/PhJ (RRID:IMSR_JAX:003715) wild-derived inbred strain also has a relatively long maxilla, but a proportionally long upper jaw for its small body size (Percival et al., 2016), allowing a test of whether these NOD/ShiLtj-like bone length proportions are the result of similar palatal segment growth trajectories. One possible mechanism for the C57BL/6J strain’s relatively short adult maxillary bone might be a slower rate of anterior secondary palate elongation between E11.5 and E15.5. If this type of variation in early A-P elongation of longitudinal palatal shelf segments is the causal basis for strain specific differences in upper jaw morphology, we predicted that C57BL/6J mice would have a relatively long primary palate and a relatively short anterior secondary palate by E15 and that the other two strains would both display relatively long anterior secondary palates at the same developmental stage.

Understanding how genetically based differences in early craniofacial development produce morphological variation within model organisms is an important step in determining how these developmental processes can be modified to produce evolutionary differences in upper jaw morphology (Boughner et al., 2008; Hallgrímsson et al., 2009; Fish, 2019; Morris and Abzhanov, 2021), including facial length and the relative contribution of specific bones to the upper jaw. Overall, our multifaceted analysis of ontogenetic trends in palatal and facial elongation provides a novel contextual framework and developmental perspective within which to evaluate the impact of both non-pathogenic and pathogenic genetic differences on midfacial growth and differentiation.

## Methods

### Specimen and Image Acquisition

**Figure 2 -:**
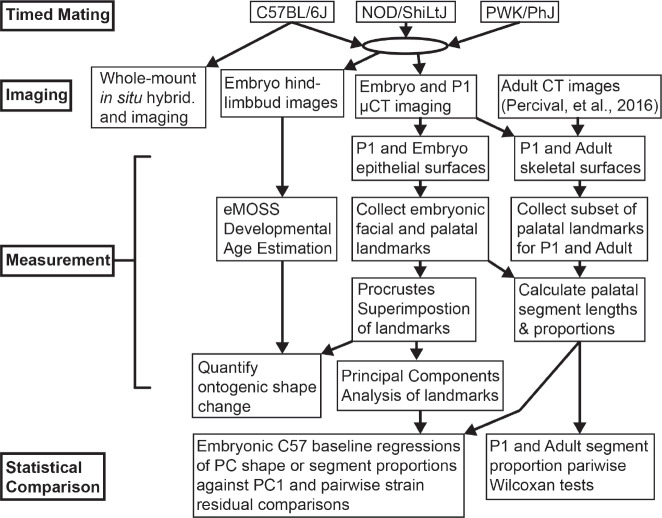
Flowchart illustrating this study’s research design

Animal breeding, specimen collection, and tissue fixation were performed in accordance with the protocols of the University of California, San Francisco Institutional Animal Care and Use Committee under protocol approval number AN192776–01F. Mice were socially housed under a twelve-hour light-dark cycle with food and water *ad libitum*. Additional enrichment was provided when single housing was required for breeding purposes. Mice were euthanized by CO_2_ inhalation followed by cervical dislocation or decapitation. Embryos of C57BL/6J (RRID:IMSR_JAX:000664; hereafter referred to as C57), NOD/ShiLtJ (RRID:IMSR_JAX:001976; hereafter referred to as NOD), and PWK/PhJ (RRID:IMSR_JAX:003715; hereafter referred to as PWK) strains (Jackson Labs, Bar Harbor, ME) were collected between gestational days E11.5 and E15.5, as determined from copulatory plug occurrence. Postnatal day one (P1) C57 (n=10) and NOD (n=7) specimens were collected one day after birth. Embryo and P1 specimens were fixed in 4% PFA and stored in 1x PBS for micro-computed tomography (μCT) imaging or dehydrated through a graded PBST-MeOH series and stored at −20°C until use for in situ hybridization. A flowchart provides an overview of our study design (Fig. 2).

### In Situ Hybridization

In situ hybridization was performed as described in Welsh et al., 2018. cDNA probes for *Phospho1* (nucleotides 274–1739 of RefSeq NM_153104), *Runx2* (nucleotides 1275–2704 of RefSeq NM_001271627), *Shh* (full length cDNA clone from A. McMahon, University of Southern California, Los Angeles CA, USA), *Shox2* (nucleotides 870–1603 of RefSeq NM_130458), *Sp7* (nucleotides 431–1715 of RefSeq NM_130458), *Tbx22* (nucleotides 307–1547 of RefSeq NM_013665) were linearized and in vitro transcribed to label with either digoxygenin (DIG) or dintrophenol (DNP). Colorimetric detection of probes used BM purple (dark blue), BCIP (teal), or MagentaPhos (magenta). Minimally, 3–6 embryos per time point were processed for each probe analyzed.

### DMDD HREM Data

High resolution episcopic microscopy (HREM) data (Geyer et al., 2009) was generated as part of the deciphering mechanisms of developmental disorders (DMDD) program (Wilson et al., 2016) and is available from the BioImage Archive at: https://www.ebi.ac.uk/biostudies/bioimages/studies/S-BIAD490?query=DMDD

### Embryonic Palatal Segment Growth Trajectories

#### Developmental Age Estimation

Given that common mouse strains vary in gestation length and there is variation in developmental timing within litters, it was necessary to standardize our cross-strain morphometric analysis by embryonic developmental age rather than gestational age. Developmental age was estimated for each μCT scanned embryonic specimen using eMOSS, an application that predicts developmental age from hindlimb bud outlines, based on a previous analysis of C57 mice (Musy et al., 2018). The resulting limb-based estimates of developmental age were reported as days since conception, up to two decimal places. We combined similar developmental age estimates whole- or half-day developmental age categories that include specimens within 0.25 days of their initial eMOSS estimate (Table 1).

Additional error may be expected when estimating the developmental age of NOD and PWK embryos using a predictive model built on C57 strain limb bud ontogeny, given the possibility of differences in the correlation of limb and head development between strains. Therefore, we also utilized the first principal component of embryonic head shape variation (PC1), based on the landmark analysis described below, as a proxy for developmental age in some analyses. PC1 score consistently tracked ontogenetic change in facial and palatal shape across strains (see Results).

#### Anatomical Landmark Collection

**Figure 3 –:**
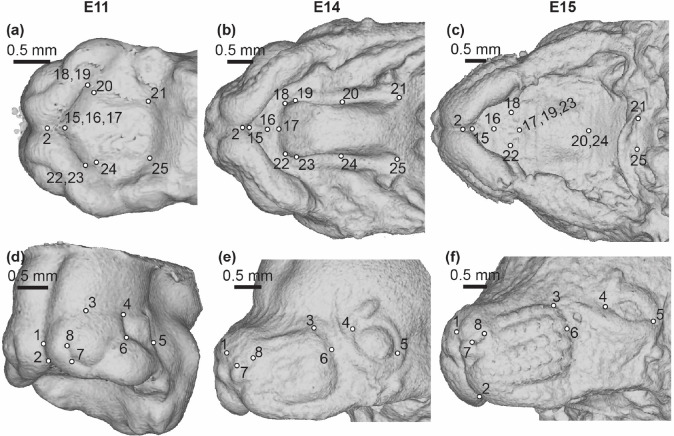
Anatomically homologous epithelial landmarks - Landmarks collected for the quantification of midfacial and palatal shape across limb based embryonic (E) developmental stages, from (a-c) palatal and (d-f) oblique facial views. Scale bars: 0.5mm. See also Supporting Table 1.

All embryo facial and palate landmarks were collected within Meshlab (Cignoni et al., 2008) on minimum threshold-based superficial tissue surfaces (downsampled x2) produced from the μCT images. Landmarks that could be identified consistently in anatomically homologous positions across the embryonic period of facial growth were chosen around the nose and whisker region, the eyes, and along the palate (Fig. 3, Supporting Table 1). The facial landmarks we collected were previously defined to track developmentally homologous structures across multiple stages of facial development (Percival, et al., 2014) and our new set of palatal landmarks were defined similarly. Similar care was taken to define embryonic epithelial tissue palatal landmarks that could be associated with the bony morphology of the P1 and adult mouse upper jaw.

#### Prenatal Geometric Morphometric Analysis

A Procrustes superimposition-based geometric morphometric analysis was used to quantify the ontogenetic shape change of the palate and face for the embryonic C57 sample and deviations of the NOD and PWK strains from this C57 baseline. The C57 strain was chosen as a baseline because it is the most widely utilized inbred strain for genetic analysis and because it was the strain originally used to train the eMOSS limb bud staging algorithm (Musy et al., 2018). Embryonic specimens with limb-based developmental age estimates between E11 and E15 were included in the embryonic shape analysis (Table 1). We performed a geometric morphometric analysis of facial landmarks using *geomorph* (Adams et al., 2020) and RRPP (Collyer and Adams, 2018) libraries in R Statistical Software (R Core Team, 2021)_._ Generalized Procrustes analysis (GPA) aligned specimen landmark sets by translating, scaling, and rotating their landmark coordinates (reviewed by Zelditch et al., 2012). Embryonic specimen shape analyses were completed using the symmetric component of Procrustes-aligned specimen landmark coordinate variation, assuming that most bilateral shape differences between the left and right sides of a specimen’s face are due to random effects associated with developmental noise and tissue fixation (Palmer and Strobeck, 1986). Thus, symmetrized landmark coordinate data were interpreted to represent the facial shape defined by a given inbred strain genotype.

The mean facial shape of specimens within each developmental age category were estimated for each strain. Differences between C57 strain age-specific mean shapes were plotted to illustrate typical facial/palatal shape growth trajectories during this important period of secondary palate elongation and midline fusion. A principal components analysis (PCA) was completed to identify the major axes of shape covariation across the embryonic sample. As with most PCAs of ontogenetic series, the first principal component (PC1) was strongly associated with overall specimen size and specimen developmental age. Therefore, we treated it as a proxy for ontogenetic growth and developmental age. C57-specific regressions represented a baseline of overall facial/palatal shape change to which the NOD and PWK specimens were compared. Due to clear inflection points in the slopes of embryonic specimen PC scores when plotted against PC1, segmented linear regressions were completed separately for PC2, PC3, and PC4 versus PC1 values for the C57 sample.

After removing three NOD specimens with PC1 scores that were substantially lower than the minimum C57 PC1 score that the C57 regression was based on, we predicted PC2, PC3, and PC4 scores from each C57, NOD, and PWK specimen’s PC1 score based on the C57 baseline regressions. Then we calculated the PC score residuals of each specimen as the difference between predicted and measured PC scores. We completed Wilcoxon rank sum tests to identify significant genotypic differences for PC2, PC3, and PC4 residual values representing the whole embryonic period at once (i.e. statistical tests were not performed at specific embryonic ages within the sampled age range). A significant difference in mean PC score residual values between strains was interpreted as a significant difference in the ontogeny of facial shape for that pair of strains.

#### Prenatal Palate Segment Length

**Figure 4 –:**
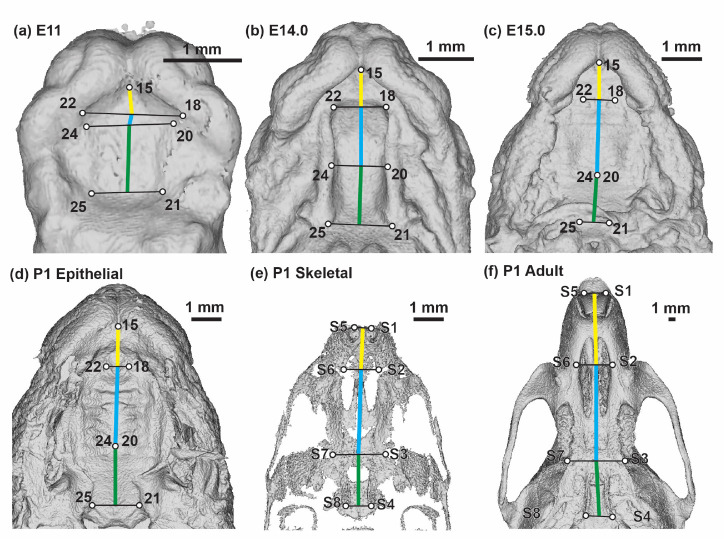
Palate segment length measurements – Identification of landmarks used to estimate midline lengths of the primary palate (yellow), anterior secondary palate (blue), and posterior secondary palate (green) on (a) E11.0, (b) E14.0, (c) E15.0 epithelial, (d) P1 epithelial, (e) P1 skeletal (after removing some neurocranial and facial bones to help with visualization), and (f) adult skeletal surfaces. Scale bars: 1mm. See also Supporting Tables 1 and 2.

The anterior-posterior length of the three major palatal segments (i.e., primary palate, anterior secondary palate, and posterior secondary palate) were estimated in millimeters from landmark coordinates without superimposition or scaling. To estimate the length of these segments along the anterior-posterior axis of the palate, the mean midline position between bilateral landmark pairs were calculated. Proportional palatal segment lengths were calculated as the length of a single segment divided by the sum of all three segment lengths for a given specimen (Fig. 4).

Small strain-specific sample sizes at multiple limb-based developmental ages meant it was not statistically appropriate to compare proportional palate segment contributions between inbred strains at single developmental ages. Instead, second-degree polynomial regressions of proportional palate segment lengths versus specimen facial shape PC1 score were calculated for the entire C57 embryo sample. Because PC1 score was considered a proxy for developmental age, these regressions define ontogenetic trajectories of palatal segment contributions to overall palatal length, which were used as a baseline for comparison with NOD and PWK proportional segment lengths. After removing three NOD specimens with PC1 scores that were substantially lower than the minimum C57 PC1 score that the C57 regressions were based on, we predicted the proportional length of each palatal segment for all specimens based on the C57 regressions. The residual differences in proportional palatal segment lengths between predicted and measured values representing the whole embryonic period at once were compared across the three strains using Wilcoxon rank sum tests. Statistical tests were not performed at specific embryonic ages within the sampled age range. A significant difference in mean residual values was interpreted to indicate a higher or lower proportional contribution of a given palate segment to overall palatal length, either across the entire embryonic period or during some major portion of this embryonic period.

### Postnatal Palate Segment Length Comparisons

To determine if embryonic differences in proportional palate segment length remained consistent postnatally and to investigate the degree of correspondence between surface epithelial and skeletal measures of palatal variation, postnatal specimen landmarks were collected on minimum threshold-based surfaces produced from 𝜇CT scans. Superficial epithelial surfaces of P1 specimens were produced using Amira (downsampled x2), and epithelial landmarks (Supporting Table 1) were collected within Meshlab (Cignoni et al., 2008).

Minimum threshold-based skeletal surfaces of P1 and adult specimens were produced using 3D Slicer (Fedorov et al., 2012) after Gaussian blur image filtering (sigma set to 0.01 for P1; sigma set to 0.02 for adult). We identified skeletal anatomical landmarks that most closely matched the palate segment landmarks defined on surface epithelium (Supporting Table 2). Landmarks were collected for C57 (n=10) and NOD (n=8) P1 specimens and for C57 (n=20), NOD (n=18), and PWK (n=18) adult specimens. Midline palate segment lengths and associated upper jaw bone lengths were measured from mean midline positions of bilateral landmark pairs without scaling or superimposition. Proportional palate segment lengths were calculated as individual segment lengths divided by sum of the three midline palatal segment lengths (Fig. 4). Wilcoxon rank sum tests were used to identify significant differences in proportional palate segment lengths between strains within single age and tissue type categories.

## Results

### Secondary Palate A-P Growth and Ruga 1’s Position at a Facial Growth Center

**Figure 5 –:**
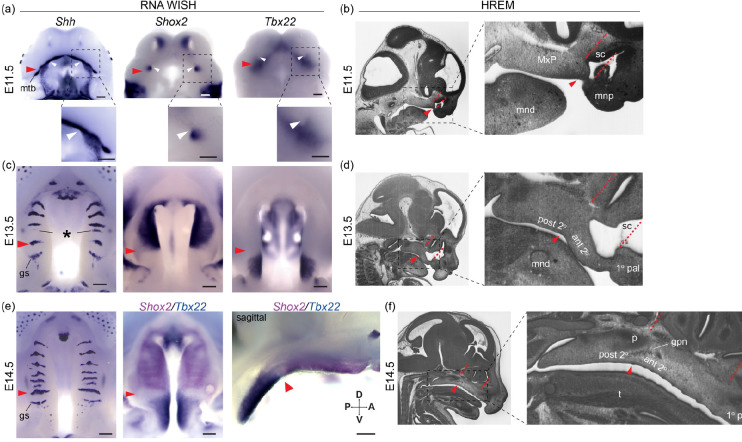
A-P molecular heterogeneity and anatomical relationships during secondary palate morphogenesis - (a, c, e) RNA WISH for *Shh*, *Shox2*, and *Tbx22* expression, and (b, d, f) sagittal sections of wildtype embryos imaged with high resolution episcopic microscopy (HREM) to provide histological resolution of the primary (1° pal), anterior secondary (ant 2°), and posterior secondary palate (post 2°) relative to surrounding facial structure during midfacial outgrowth at (a, b) E11.5 (c, d) E13.5, and (e, f) E14.5. Throughout midfacial outgrowth, ruga 1 (red arrowheads) marks the shared A-P expression boundary of *Shox2* and *Tbx22* in the anterior and posterior secondary palate, respectively. Double label WISH for *Shox2* (magenta) and *Tbx22* (blue) at E14.5 highlights mutually exclusive anterior and posterior expression domains organized relative to ruga 1 (oral view left, sagittal view right). White arrowheads mark the choanae, black asterisk marks the RGZ. Red dashed lines mark position of coronal planes passing through the primary-secondary palate junction and posterior wall of the nasal capsule to highlight coordinated elongation of the anterior secondary palate and overlying sinus cavity (sc). Regions in dashed boxes of (a) and in HREM images shown enlarged below or to the right, respectively. Additional abbreviations: mandible (mnd), molar tooth bud (mtb), maxillary process (MxP), medial nasal process (mnp), tongue (t), greater palatine nerve (gpn), geschmacksstreifen (gs), palatine (p), posterior domain of *Shh* expression (pd). Scale bars: 250um

Close relationships between different anatomical domains underlying midfacial outgrowth are found centered near ruga 1 ( & Supporting Fig. 1, red arrowheads). Our embryonic series confirmed that ruga 1 formed within the MxP from a domain of *Shh* expression at the anterior extreme of the nascent secondary palate. As previously shown, other rugae then formed anterior to ruga 1 at the RGZ (Fig. 5c, asterisk) as the anterior secondary palate elongated, anteriorly displacing the primary palate and external nares. A separate domain of *Shh* expression posterior to ruga 1 gave rise to the geschmacksstreifen (gs) and sensory papilla arrayed across the most posterior palate. Our embryonic series also confirmed that ruga 1 is consistently found at the *Shox2/Tbx22* gene expression boundary between the anterior and posterior secondary palates during this critical embryonic period of palatal development (Fig. 5 & Supporting Fig. 1).

We identified a previously underappreciated association between ruga 1 and the posterior wall of the nasal capsule. At E11.5, ruga 1 formed adjacent to the primary choanae (Fig. 5a & Supporting Fig. 1a, white arrowheads); the posterior openings of the nasal passages, which initially occur at the boundary of primary and secondary palatal segments (Tamarin, 1982). Elongation of the anterior secondary palate then coincided with the formation and expansion of the overlying nasal capsule. As the primary choanae and nasal capsule elongated between E11.5 and E15.5, ruga 1 and the posterior wall of the nasal capsule remained in approximately the same coronal plane (represented by position of red dashed lines in Fig. 5b, 5d, 5f and Supporting Fig. 1). Ruga 1 was also found coincident with a gross morphological inflection point of the palatal tissue where the anterior secondary palate and overlying nasal capsule exhibited an inferiorly accentuated angle relative to the cranial base, while the posterior secondary palate did not (Fig. 5d–f, Supporting Fig. 1).

The greater palatine neuro-vascular bundle was also consistently associated with ruga 1 across this embryonic period. The greater palatine nerve and artery were found entering the palatal shelves from a location immediately dorsal to ruga 1 at E12.5 and this spatial relationship was maintained until the greater palatine foramen formed by the articulation of maxilla and palatine bones around the palatine neuro-vascular bundle at the maxillary-palatine suture (Fig. 5f, Supporting Fig. 1). In combination, the consistent associations between ruga 1, the anterior-posterior secondary palate boundary, the posterior margin of the nasal capsule, and greater palatine neurovascular bundle suggested that these features are positioned at a proximal source of directional growth that the elongating anterior secondary palate and nasal capsule extend away from between E11.5 and E15.5.

**Figure 6 –:**
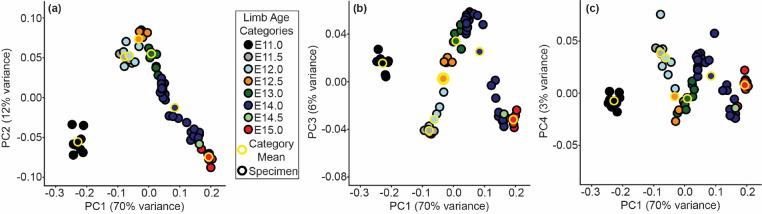
Major axes of embryonic facial shape variation - C57 embryo specimens plotted along the (a) first and second principal component axes of shape (i.e., PC1 and PC2), (b) along PC1 and PC3, and (c) along PC1 and PC4. Circle color indicates the limb based developmental age categories of each specimen, with the yellow bordered circles indicating the mean PC scores for the C57 mice of that age category. The proportions of facial shape variance associated with each principal component are provided. The principal component axes were estimated from the full sample of E11-E15 developmental age embryos, including C57, NOD, and PWK samples, although only C57 samples are plotted in this figure. The same plot, but incorporating all three inbred strains is available as Supporting Figure 2.

**Figure 7 –:**
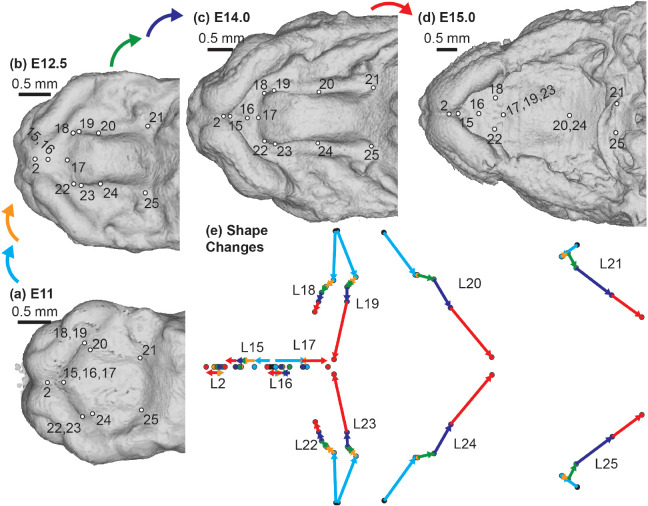
C57 palatal landmark growth trajectories – The positions of palatal landmarks are identified on representative C57 specimens at limb-derived developmental ages (a) E11, (b) E12.5, (c) E14, and (d) E15. (e) The average palatal landmark positions are plotted for each developmental age category that had more than one C57 specimen. These landmark positions represent palatal shape after the removal of overall facial scale during Procrustes superimposition. The arrows indicate the trajectory of shape change for each landmark between ages. Black=E11; Light Blue = E12; Orange = E12.5; Green = E13; Dark Blue = E14; Red = E15. Scale bars: 0.5mm

A complementary 3D morphometric analysis of midfacial and palatal epithelial landmarks was used to quantify the integrated morphogenetic processes occurring during this critical developmental period. The first major principal axis of facial shape variation (PC1) for all limb bud staged E11-E15 specimens represented 70% of shape variance and was associated with general ontogenetic growth during this time window, as illustrated for the C57 sample (Fig. 6) and for all three inbred strains (Supporting Fig. 2). Additional principal axes of variation for C57 embryos suggested shifts in the direction of ontogenetic shape changes over developmental time, with one inflection noted along PC2 (Fig. 6a), two along PC3 (Fig. 6b), and three along PC4 (Fig. 6c). Average developmental age category shape differences were illustrated as landmark specific ontogenetic shifts in palate shape (Fig. 7) and overall midfacial shape (Supporting Fig. 3).

Between developmental stages E11 and E15, each of the three major palatal segments grew in length, but the anterior secondary palate grew proportionally more than the primary palate and posterior secondary palate (Figs. 5 & 7). The primary palate represented ~20% of the total palate length, the anterior secondary palate ~20%, and the posterior secondary palate ~60% for C57 embryos soon after the start of A-P palate elongation. After E12.5, secondary palate length (relative to overall facial size) increased noticeably with each developmental stage. By the time of midline palatal fusion, the primary palate represented ~25% of palate length, the anterior secondary palate represented >40%, while the posterior secondary palate represented <35%. Therefore, between E11 and E15, the proportional contribution of the primary palate increased by a quarter while posterior secondary palate contribution decreased by nearly a third. Strikingly, the proportional contribution of the anterior secondary palate to overall palate length doubled during this period.

### Segmental Differences in Skeletal Specification, Differentiation, and Growth During Secondary Palate Morphogenesis

We performed a WISH analysis of *Runx2*, *Sp7*, and *Phospho1* expression () alongside double labeled WISH of *Shh* and *Sp7* between E12.5 and E15.5 () to characterize bone specification and differentiation in relation to A-P growth dynamics of secondary palate segments. The position of rugae, which provided an ordered set of temporally specific anatomical features, allowed for the identification of previously unappreciated associations between the palatal segments and developing palatal bones.

**Figure 8 –:**
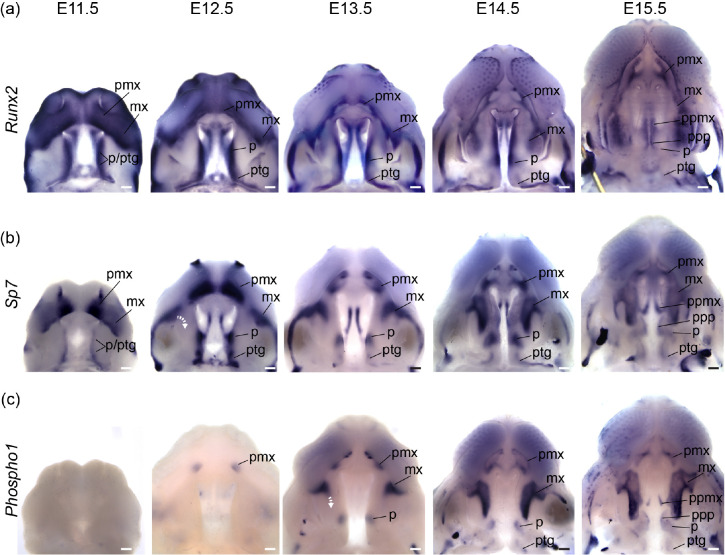
Segmental organization of skeletal specification and growth during A-P morphogenesis of the midface – WISH time course for (a) *Runx2*, (b) *Sp7*, and (c) *Phospho1* between E11.5 and E15.5. Expression of *Runx2* in osteoprogenitors highlights facial domains with osteogenic potential during midfacial morphogenesis. Expression of *Sp7* and *Phospho1* in committed and differentiating osteoblasts delineates the growth dynamics of individual skeletal anlagen during midfacial morphogenesis. Curved white dashed arrows in (b) and (c) indicate medial expansion of maxillary anlage. Abbreviations: maxilla (mx), palatine (p), premaxilla (pmx), pterygoid (ptg), palatal process of the maxilla (ppmx), palatal process of the palatine (ppp). Scale bars: 250um

Broad *Runx2* expression in early facial prominences included osteogenic domains (e.g., premaxilla within the primary palate) and later became more restricted to regions adjacent to the molar tooth bud and medially along the adjoining palatal shelves (Fig. 8a). Osteoblast expression of both *Sp7* and *Phospho1* more precisely delineated the morphology of the emerging midfacial skeleton (Fig. 8b–c). Palatal processes of the maxilla and palatine bones formed within the medial region of the elevated palatal shelves, a domain that maintained high levels of *Runx2* expression during secondary palate growth (Fig. 8a), suggesting maintenance of a less differentiated osteoprogenitor population until palatal elevation and fusion was complete.

At E11.5, *Sp7* was strongly expressed in bilateral domains within the mnp of the forming primary palate. Between E12.5 and E15.5, presaging premaxilla morphology, *Sp7* expression formed a cup-like region surrounding the developing incisors (Fig. 8b). *Phospho1* was first observed in the forming premaxilla at E12.5 (Fig 8c), reflecting a temporal lag between initial osteoblast commitment and later differentiation. The premaxilla acquired its characteristic adult morphology within the primary palate segment in which it was initially specified.

Within the secondary palate, this simple developmental sequence was repeated for the palatine and pterygoid bones within the posterior secondary palate, but development of the maxilla within the anterior secondary palate was unique. At E11.5, *Sp7* expression in the MxP was observed in two domains. The anterio-lateral domain, which abutted the point of fusion with the mnp (i.e., the primary palate) and extended laterally away from the oral cavity and choanae, corresponded to the maxillary anlage. The posterior domain that spanned the length of the forming secondary palate, from the anterior limit abutting the choanae to the posterior-most palatal edge, gave rise to the palatine and pterygoid anlagen.

**Figure 9 –:**
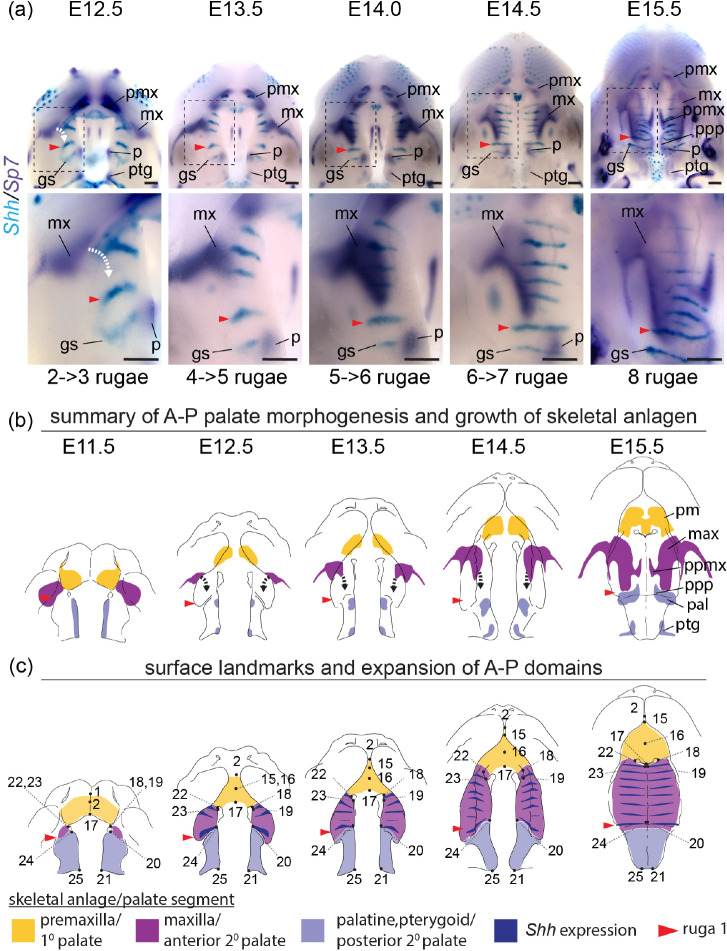
Double label RNA WISH time course for *Shh* (cyan) and *Sp7* (dark purple) between E12.5 and E15.5 - (a) Expression of *Sp7* in committed and differentiating osteoblasts delineates the growth dynamics of individual skeletal anlagen and *Shh* expression in rugae provides a temporally ordered set of A-P landmarks (regions in dashed boxes enlarged below). (b) Summary model of skeletal growth dynamics during midfacial outgrowth. Growth of the premaxilla (yellow) and palatine (pale blue) anlagen towards their characteristic shape occurs largely at the site of initial specification. Following initial specification external to the oral cavity, the maxilla (purple) grows into the anterior of the anterior secondary palate (curved white and black dashed arrows in a and b, respectively) towards the position of ruga 1 (red arrowhead) and palatine as expansion of the anterior secondary palate (double headed red arrow) separates the primary and posterior secondary palate. (c) Summary of the position of epithelial landmarks (see also Fig. 3) selected to capture segmental growth dynamics of the primary palate (yellow) and anterior secondary palate (purple) and posterior secondary palate (pale blue) during midfacial outgrowth. Abbreviations: geschmacksstreifen (gs), maxilla (mx), palatine (p), premaxilla (pmx), pterygoid (ptg), palatal process of the maxilla (ppmx), palatal process of the palatine (ppp). Scale bars: 250um

The antero-lateral domain of *Sp7* expression adjacent to the primary palate was initially external to the secondary palate at the site of the future zygomatic plate. However, this expression domain later expanded medially (E12.5-E13.5) and then posteriorly (E13.5-E15.5) within the growing anterior secondary palate (white curved dashed arrows in Fig. 8b). This expansion occurred after initial A-P growth of the anterior secondary palate and formation of 3–4 rugae anterior to ruga 1, suggesting that it is coupled with RGZ growth dynamics (Fig. 9a). The fact that *Phospho1* expression closely followed *Sp7* spatial dynamics further supported the idea that these *Sp7* expression patterns represent maxillary bone anlagen formation and expansion (white curved dashed arrow in Fig. 8c). The maxillary anlage continued growing posteriorly towards ruga 1 concomitant with rugae formation (white and black curved dashed arrows in Fig. 9a–b), within the elongating secondary palate.

The posterior domain of *Sp7* expression that initially spanned the length of the secondary palate at E11.5 was displaced posteriorly by expansion of the anterior secondary palate between E12.5 and E15.5. During this period, it separated into two subdomains. The anterior subdomain remained associated with the posterior wall of the nasal capsule and gave rise to the palatine bone, while the posterior subdomain formed both the medial and lateral pterygoid processes (Fig. 8b–c). Ruga 1 and the palatine anlage initially formed at the anterior extent of the secondary palate at E11.5 and maintained proximity to each other throughout palatal development even as both structures were displaced posteriorly (Fig. 9a).

The palatine and pterygoid anlagen grew to acquire their characteristic adult morphology within the posterior secondary palate segment where their precursor domains were initially specified, mirroring premaxillary anlagen formation within the primary palate. This differs from growth of the maxillary anlage that was initially specified external to the secondary palate at the site of the future zygomatic plate and only later extended into the anterior secondary palate, following a period of initial elongation, to give rise to the majority of the maxillary bone.

These results indicated that the posterior portion of the secondary palate and its associated anlage were present within the secondary palate at the onset of its morphogenesis at E11.5, while the anlage associated with the anterior secondary palate (i.e. the maxilla) did not contribute substantially to the secondary palate until later (Fig. 9b). They also indicated that the eventual meeting of the maxilla and palatine anlagen was an asymmetric process achieved predominantly by posterior growth of the maxilla towards the palatine (white and black curved dashed arrows in Fig. 9-b). By E15.5, the maxilla and palatine anlagen met to form the maxillary-palatine suture subjacent to ruga 1 (see also Supporting Fig. 1).

Our results collectively revealed that the segmental relationship between three palatal regions and associated bone anlagen remained consistent throughout the period of palatal elongation, although the maxillary anlage was not present within the anterior secondary palate until part way through this period. Importantly, these results also highlighted the previously unappreciated correlation of maxillary growth to rugae formation as well as to the greater proportional expansion of the anterior secondary palate between E11.5 and E15.5 (summarized in Fig 9C).

### Interstrain Comparisons of Facial and Palatal Growth

We completed a morphometric comparison of C57, NOD, and PWK strain embryonic midfacial shapes and a comparison of proportional palatal segment lengths across embryonic and postnatal stages to test the hypothesis that early differences in palatal segment growth contribute to variation in adult midfacial shape, including strain specific differences in proportional bone contributions to upper jaw length.

Because developmental speed varies between mouse strains and within litters, we standardized our cross-strain ontogenetic analysis using limb bud outline-based embryonic developmental age rather than gestational age. Limb bud developmental ages correlated strongly with the number of days after plug identification for each strain. Though the correlation coefficients for C57 mice (0.98), NOD (0.93) and PWK (0.90) were high, there was a larger difference between these two embryonic age estimates for our PWK samples. The C57 specimen limb-based developmental ages were an average of 0.26 days younger than our copulatory plug estimates, while NOD and PWK limb ages were an average of 0.30 days and 1.09 days younger, respectively. The major divergence for PWK was likely driven by a slower speed of embryonic development in this wild-derived strain compared to the two common lab strains (Murray et al., 2010). Even so, standardization by developmental age should allow for improved direct comparisons between embryos of all strains.

**Figure 10 –:**
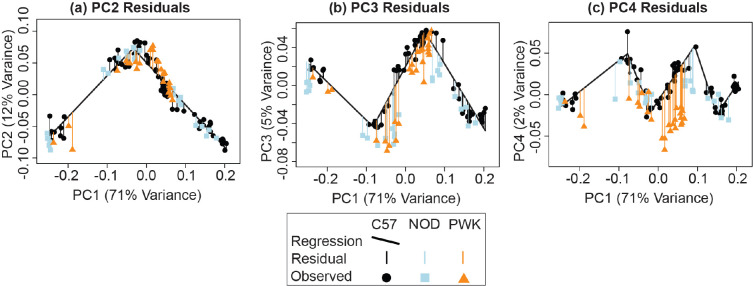
Specimen PC score residuals – The principal component (PC) score residuals for each specimen, relative to the scores predicted from C57 strain specific segmented linear regressions of (a) PC2, (b) PC3, and (c) PC4 on the specimen’s PC1 score. In this way, the C57 facial shape growth trajectory serves as a baseline with which to compare the shape of NOD and PWK specimens, based on the fact that the PC1 axis is strongly associated with facial shape ontogeny from developmental age E11 to E15.

We compared the midfacial shape of NOD and PWK specimens with C57 specimens along the principal axes of facial shape variation. This comparison indicated that mice with similar limb-based developmental age estimates tended to fall near each other along the first and second principal components, regardless of strain. As these axes represented 82% of facial shape variance across our sample, all mouse strains displayed major similarities in ontogenetic shape change across this period (Supporting Fig. 2a). However, across ontogenetic time (as represented by PC1 score) NOD and PWK specimens had significantly lower PC3 values compared to the C57 baseline, based on a comparison of regression residual values representing the entire sampled embryonic period (Fig. 10b). PWK specimens also had significantly lower PC4 values (Fig. 10c).

**Figure 11 –:**
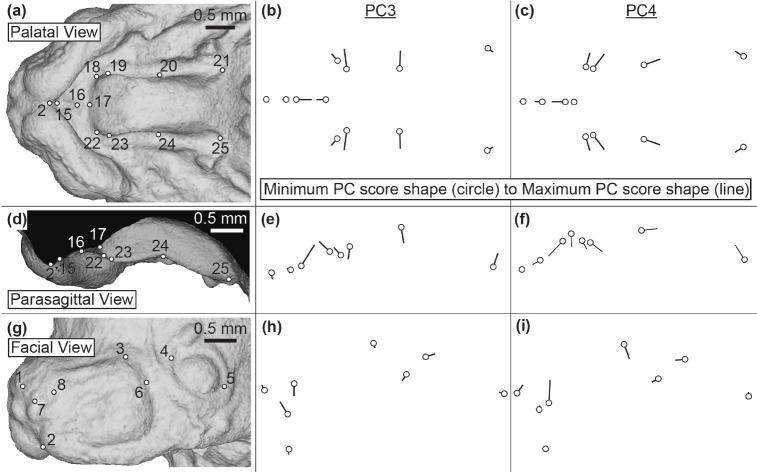
Facial shape variation associated with PC3 and PC4 – Representation of the pattern of shape variation associated with the third major axis and fourth major axis of embryonic facial/palatal shape variation, from (a-c) palatal, (d-f) parasagittal, and (g-i) facial views. Superficial tissue landmarks are identified on an example E14.5 specimen to assist with interpretation of the landmark vectors that represent differences in minimum (circle) and maximum (end of line) PC scores. See Supporting Fig. 2 for a plot of embryonic specimens along these major axes of embryonic shape variation. Scale bars: 0.5mm

We visualized how these identified significant differences in PC3 or PC4 scores were reflected within facial and palatal morphology by comparing shapes represented at the minimum and maximum end of these major shape axes (Fig. 11), with strain representative whole mount (Supporting Fig. 4) and 𝜇CT surface (Supporting Fig. 5) images provided for reference. A minimum PC3 score was associated with the two nostril landmarks (LM7 & 8) being relatively distant in the superior/inferior and lateral directions. Lower PC3 score was also associated with relative proximity of the posterior whisker margin (LM6) to the anterior canthus (LM4) (h). Within the palate, lower PC3 scores were associated with a relatively medial anterior secondary palatal shelf, suggesting more medial palatal outgrowth (proportional to overall palatal length) at a given developmental stage (Fig. 11b). The superior-inferior position of the palatal landmarks indicated that the secondary palate was more highly angled (less flat) for specimens with low PC3 scores (Fig. 11e). Because of their generally lower PC3 scores, we anticipated more vertical nostrils in NOD and PWK mice with more highly angled palatal shelves in greater medial proximity to each other, when compared to C57 mice within a given developmental age.

A minimum PC4 score was associated with the whole nasal region (LM1, LM8) being positioned more inferiorly and the posterior edge of the whisker region (LM3, LM6) more superiorly when compared to the other facial and palatal features (Fig. 11i, Supporting Figs. 4 & 5). Within the palate, a low PC4 score was associated with a relatively anterior location of ruga 1 between anterior and posterior secondary palate (LM20 & 24) and a relatively posterior position of the posterior edge of the posterior secondary palate (LM21 & 25 within Fig. 11c). Low PC4 scores were also associated with more medially positioned anterior secondary palate landmarks that are closer to the boundary with the primary palate. Because of their generally lower PC4 scores, this suggested that PWK embryos display a relatively low superior nasal region, a medially expanded anterior secondary palatal shelf with a relatively short anterior secondary palate and relatively long posterior secondary palate, when comparisons are made within a given developmental age.

**Figure 12 –:**
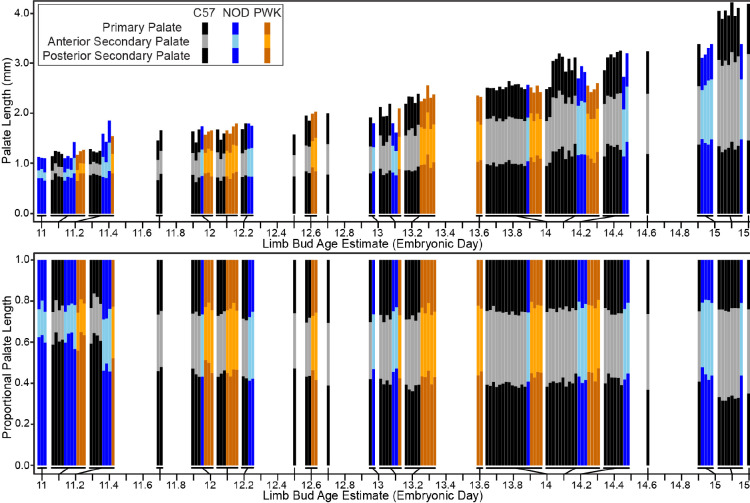
Embryonic palate segment length across ontogeny – The midline projected strain-specific mean lengths of the primary palate, anterior secondary palate, and posterior secondary palate, for all E11-E15 specimens binned into 0.1 day limb bud derived developmental age categories and sorted by genotype. Raw length values (in millimeters) are presented above, while palatal segment length relative to overall midline palate length are presented below.

**Figure 13 -:**
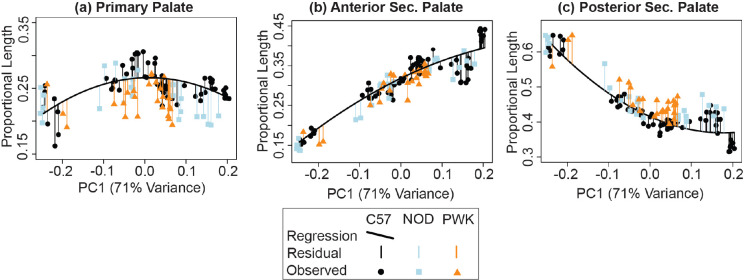
Specimen palate segment length residuals – The proportional contributions of each palate length segment (shapes), as compared to the proportional contributions predicted from C57 strain specific linear regressions (curved lines) of proportional (a) primary palate, (b) anterior secondary palate, and (c) posterior secondary palate midline lengths versus the specimen’s PC1 score. Vertical lines represent the residual of each specimen from C57 regression. In this way, the C57 palatal segment growth trajectory serves as a baseline that NOD and PWK specimens were compared to, based on the fact that the PC1 axis is strongly associated with facial shape ontogeny from E11 to E15.

A comparison of embryonic palatal segment lengths (Fig. 4a–c) across C57, NOD and PWK samples indicated that all three mouse strains display parallel ontogenetic changes 1) in overall palatal length and 2) in the proportional contributions of palatal regions to overall palatal length during the period of early palatal morphogenesis (, Supporting Fig. 6). This includes generally increased proportional contributions of the anterior secondary palate and decreased proportional contributions of the posterior secondary palate between E11 to E15, as described above for C57 mice. However, small but significant differences in palatal segment proportions between mouse strains were identified within a comparison of proportional palatal segment length versus facial shape PC1 score (a proxy for developmental age). These comparisons were completed based on regression residuals representing the entire sampled embryonic age range and were not completed at specific embryonic ages. The NOD and PWK primary palate proportional length residuals were significantly lower than for C57 (p-values: 0.012 (NOD vs C57), <0.001 (PWK vs C57)) (Fig. 13a), while they also had significantly higher posterior secondary palate residuals than C57 (p-values <0.001) (Fig. 13c). This indicated that the primary palate contributed relatively less and the posterior secondary palate contributed proportionally more to total embryonic palate length in these two mouse strains, although visual assessment suggested these differences may not be apparent at the earliest measured developmental ages (Fig. 12) or for specimens with the lowest PC1 scores (Supporting Fig. 6). Significantly lower anterior secondary palate residuals for NOD and PWK (p-values: 0.029 (NOD vs C57), 0.023 (PWK vs C57)) indicated that the anterior secondary palate represented a lower proportion of the total palate length within these strains (Fig. 13b), although these differences seemed less pronounced than those noted for the other two segments (Fig. 12, Supporting Fig. 6). Taken together, these trends suggested that C57 mice typically had proportionally longer primary palates and proportionally shorter posterior secondary palates than NOD and PWK mice during this embryonic period, with a trend towards longer anterior secondary palates by the time of medial palatal shelf fusion (Fig. 13).

### Interstrain Comparisons of Postnatal Palatal Segments

**Figure 14 –:**
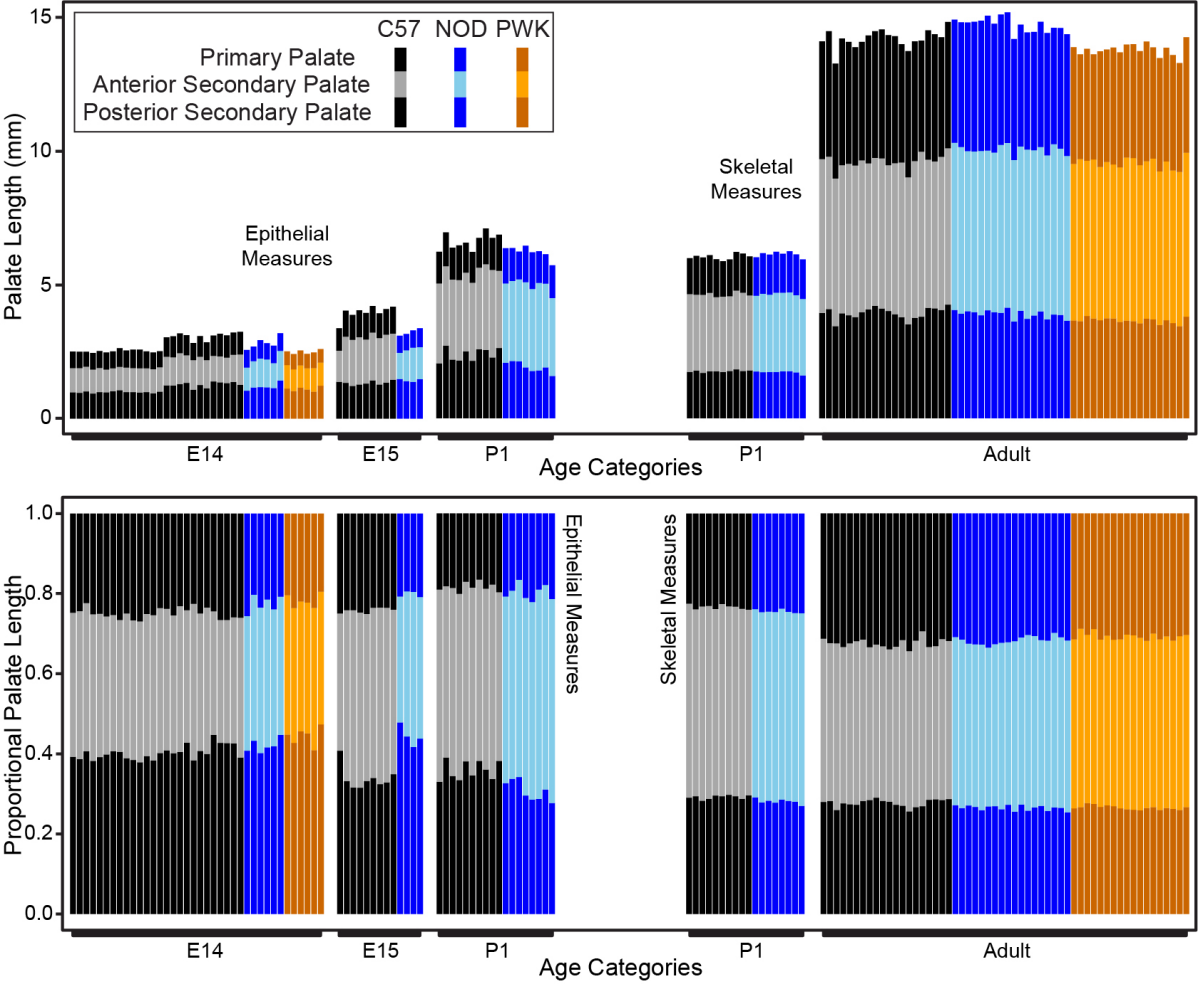
Interstrain comparisons of palate segment lengths in older specimens – The midline projected lengths of the primary palate, anterior secondary palate, and posterior secondary palate, based on epithelial landmark measures for all specimens of limb based developmental stages E14 and E15, as well as for P1 specimens. Analogous length measures of primary palate derived premaxilla, anterior secondary palate derived maxilla, and posterior secondary palate derived palatine/pterygoid bones are presented for all P1 and adult specimens. Raw length values (in millimeters) are presented above, while palatal segment length relative to overall midline palate length are presented below.

We compared C57 and NOD proportional palatal segment lengths at P1 based on epithelial surface landmarks and skeletal landmarks of associated bony elements. Based on the epithelial surface landmarks (Fig. 4d), C57 had a proportionally shorter anterior secondary palate and a proportionally longer posterior secondary palate (p-values <0.01) than NOD at P1 (Fig. 14; Table 2). This differed from the embryonic pattern where C57 specimens generally displayed proportionally shorter posterior secondary palates than NOD specimens. The significant differences in epithelially-measured proportional segment contributions between NOD and C57 mice at P1 appeared largely based on differences in the measured length (in mm) of the posterior secondary palate (Fig. 14).

A comparison of proportional palatal bone lengths in P1 samples (Fig. 4e) indicated that C57 has a proportionally shorter premaxilla (associated with primary palate) and proportionally longer palatine/pterygoid bones (associated with posterior secondary palate) (p-values <0.01) than NOD (Fig. 14; Table 2). A similar comparison of proportional palatal bone lengths (Fig. 4f) in 8–12 week old adult specimens of all three strains revealed that the premaxillary (primary palate) contribution to total palate length is significantly lower in PWK (p-value: < 0.001) and NOD (p-value: 0.044) compared to C57, with the opposite true for the maxillary (anterior secondary palate) portion of the palate (p-values: < 0.001) (Fig. 14). The palatine/pterygoid (posterior secondary palate) portion was proportionally shorter in PWK and NOD when compared to C57 (p-values: < 0.001) (Fig. 14). The PWK average proportional measures of premaxilla and maxilla showed a more extreme divergence from C57 than in the NOD mouse comparison.

Overall, there were some ontogenetic changes in how strains differed in proportional palate lengths, either between the embryonic period and P1 or between P1 and adulthood. NOD and C57 showed parallel changes in palatal bone contributions across the postnatal period, where the posterior secondary palate contribution remained fairly stable (~27–29%), but the primary palate contribution increased from ~24% to ~32% and the anterior secondary palate contribution decreased from ~47% to ~40–42% (Fig. 14; Table 2). During the postnatal period, the entire mouse and its palate grew substantially, but premaxilla growth contributed more than the maxilla growth to overall postnatal palatal elongation. Although the A-P growth of the anterior secondary palate was a critical driver for midfacial growth between E11.5 and E15.5, outgrowth of the premaxilla plays a larger role in postnatal midfacial outgrowth.

## Discussion

Our results provided a multifaceted characterization of 1) normal murine midfacial growth dynamics within the secondary palate and upper jaw bones during the earliest stages of palatal growth and 2) subsequent changes in upper jaw bone proportions in newborn and adult mice. By tracking the position of the RGZ, sequential palatal rugae formation, and palatal bone precursor populations across the earliest phases of palatal A-P elongation, our results illustrated that the first-formed palatal ruga (i.e., ruga 1), which sits at an important border of regulatory gene expression is coincident with important nasal, neurovascular and palatal structures throughout early midfacial development. For example, ruga 1 represents a consistent morphological boundary between the presumptive maxilla of the anterior secondary palate and the presumptive palatine and pterygoid bones of the posterior secondary palate. This association suggested that the process of rugae morphogenesis is coupled to maxillary osteogenesis during anterior secondary palate expansion and that ruga 1 approximates the future position of the maxillary-palatine suture from the time of its formation.

Our ontogenetic comparison of epithelial and skeletal palatal anatomy across three mouse strains identified small, but significant strain-specific differences in early palatal morphogenesis and normal midfacial outgrowth. These differences provide a useful foundation for understanding the impact of background genetic effects on facial shape and elongation. However, embryonic differences in palatal segment proportions did not match adult strain-specific differences in the contributions of the premaxilla, maxilla, and palatine/pterygoid bones to upper jaw length.

### Normal Upper Jaw and Palatal Elongation

It is well known that A-P elongation of the palate is a necessary component of the integrated processes of midfacial outgrowth. However, within mammals, we previously lacked a detailed picture of the earliest spatiotemporal dynamics of intramembranous midfacial skeletal specification and differentiation within the context of the surrounding palatal segments and rugae formation. Our combined whole mount gene expression and morphometric analysis indicated that the relationship between three longitudinal palate segments and associated upper jaw bones was already established during the earliest phases of palatal morphogenesis. These results illustrated the substantial contribution of embryonic anterior secondary palate elongation to overall palate length in mice and likely in other mammals. Anterior secondary palate elongation was also directly associated with rugae formation at the RGZ and elongation of the maxillary bone primordium.

Beyond verifying that ruga 1 forms at an important gene expression boundary between the anterior and posterior secondary palate, our results clarified that ruga 1 represented a stable morphological boundary positioned near the future maxillary-palatine suture, as opposed to the alternate hypothesis that it is positioned between hard and soft palate (Pantalacci et al., 2008). Ruga 1 formed at the earliest stages of anterior secondary palate elongation, then maintained a position at the anterior edge of the palatine bone anlage as it was posteriorly displaced by expansion of the anterior secondary palate. Anterior to ruga 1, the maxillary bone osteogenic population expanded from the site of initial specification (i.e. the zygomatic plate) into the secondary palate after ruga 1 was posteriorly displaced during anterior secondary palate elongation. Given that the secondary palate initially only contained osteogenic domains of the posterior secondary palate (palatine and pterygoid precursors), these results suggested that cellular cues in the proximity of ruga 1 may initially inhibit maxillary bone formation within the secondary palate. Additionally, expansion of the palatal portion of the maxillary anlage was uniquely associated with RGZ dynamics and anterior secondary palate elongation, suggesting that RGZ morphogenesis plays a major role in determining the proportional contribution of the maxilla to the upper jaw within a broad evolutionary context. This hypothesis could be tested within an evo-devo context by characterizing variation in palatal *Shh* expression between mammals and avians that have drastic differences in maxillary morphology, including an obligate avian cleft palate. Alternatively, it could be dissected genetically via analysis of *Shh* expression and rugae morphogenesis in mice with altered A-P skeletal patterning, such as the *Pbx* CNCC mouse mutants (Welsh et al., 2018).

Differences in upper jaw suture anatomy may also be related to segmental differences in early bone growth. The premaxilla and maxilla were specified in adjacent tissue domains that maintained close apposition throughout palate morphogenesis, suggesting equal contributions to the formation of the premaxillary-maxillary suture. Conversely, the palatal portion of the maxilla grew posteriorly towards the palatine anlage at ruga 1, suggesting that maxillary growth (and likely RGZ dynamics) played a larger role than palatine growth in determining maxillary-palatine suture position. Different growth dynamics at these sutures may lead to a more vertical premaxillary-maxillary suture versus a more oblique maxillary-palatine suture with substantial A-P overlap of the maxilla and palatine bones.

Because suture formation provides a critical niche for facial skeletal progenitors (Zhao et al., 2015), the way palatal segments contribute to palatal sutures may contribute to variation in postnatal dynamics of midfacial growth and remodeling (Enlow and Bang, 1965; Kurihara et al., 1980; Sarnat, 1997; Martinez-Maza et al., 2013; Vora et al., 2015; Maga, 2016). The widespread presence of Gli1+ mesenchymal stem cells and nascent bone within the adult maxillary-palatine suture and the maxillary bone, but not in the palatine bone (Luo et al., 2019) provides further support for this hypothesis. Given the established role of calvarial sutures in directing postnatal craniofacial bone growth, further comparisons of osteoprogenitor dynamics during premaxillary-maxillary and maxillary-palatine suture formation are warranted.

### Palatal Growth as a Basis for Morphological Variation

Given its central contribution to total palatal elongation, variation in RGZ regulated A-P growth of the anterior secondary palate may contribute substantially to the range of prognathism observed amongst mammals (Young et al., 2014). Support for this idea comes from the fact that more prognathic species tend to have more rugae: flat faced humans typically have 3–6 palatal rugae (Hauser et al., 1989; Jayasankar et al., 2016), mice have 8–9 rugae (Peterkova et al., 1987), and prognathic pigs have 20–25 rugae (Tonge and McCance, 1965). Based on 1) the expectation that interspecies differences are extensions of milder intra-species differences and 2) the direct early associations between palatal segments and upper jaw bone morphogenesis, we hypothesized that strain-specific contributions of individual bones to upper jaw length (Percival et al., 2016) would be produced by strain-specific differences in early embryonic A-P palatal segment elongation.

We identified small, but significant strain-associated differences in facial shape and palatal segment length. Embryonic facial shape differences between strains (Figs. 12 & 13; Table 2; Supporting Fig. 6) may represent ontogenetic shifts in the relationship of various facial structures or shifts in the relative timing of normal developmental events occurring within different parts of the face. Subtle differences in embryonic palatal segment proportions were identified between the strains when considering the entire embryonic series at once, but these differences did not match the inter-strain differences observed in adult specimens. Additionally, some proportional palatal contribution differences for NOD and C57 changed between E15 and P1 as well as between P1 and adult samples. A larger sample of later embryonic ages (i.e., between E15.5 and P0) would allow us to determine when the initial NOD pattern of short primary and long posterior secondary palate changes to the P1 pattern of long primary and short posterior secondary palate.

Although changes within the RGZ growth dynamics of anterior secondary palate growth may be critical for major species-specific differences in prognathism, our results did not support the idea that this mechanism is primarily responsible for the adult intraspecies differences in upper jaw morphology between our mouse strains. This suggests that later prenatal (after E15) and postnatal growth processes play a major role in determining adult upper jaw bone proportions between inbred mouse strains. Broad embryonic growth patterns shared across our strains indicated that elongation of the anterior secondary palate contributes most to palatal A-P growth between E11 and E15, but that growth of the primary palate derived premaxilla contributed most to postnatal elongation of the upper jaw.

### Concluding Statement

Our multifaceted illustration of normal midfacial growth dynamics confirmed a one-to-one relationship between palatal segments and upper jaw bones during the earliest stages of palatal growth, suggesting that the first formed ruga represents a consistent morphological boundary between anterior and posterior secondary palate bone precursors, thus approximating the position of the future maxillary-palatine suture. In addition to driving rugae formation, our results suggested that interactions at the RGZ simultaneously coordinate elongation of the maxillary bone primordium within the anterior secondary palate, which more than doubles in length prior to palatal shelf fusion. Although RGZ-driven A-P growth of the anterior secondary palate likely contributes to evolutionary changes in facial upper jaw morphology, this process was not responsible for the small but significant strain-specific differences in adult upper jaw bone proportions measured across three inbred mouse strains. However, measured differences in early palatal segment elongation provide a useful foundation for understanding the impact of background genetic effects on facial morphogenesis. Our multifaceted analysis of ontogenetic trends in palatal and facial elongation provides a novel contextual framework and developmental perspective within which to evaluate the impact of both non-pathogenic and pathogenic genetic differences on midfacial growth and differentiation.

## Supplementary Material

Supplement 1

## Figures and Tables

**Table 1 –:** Sample size of μCT imaged and morphometrically analyzed embryo specimens by strain and forelimb based developmental age estimation, as estimated using eMOSS (Musy et al., 2018).

Limb-Based Developmental Age	C57	NOD	PWK
E11	8	12	4
E11.5	2	0	0
E12	7	3	6
E12.5	4	0	2
E13	9	3	6
E13.5	0	0	2
E14	26	6	6
E14.5	1	0	0
E15	9	4	0

**Table 2 –:** Average proportional palatal segment lengths for postnatal specimens, presented as percentages of total palatal length.

	C57			NOD			PWK
	P1 epithelial	P1 bone	Adult bone	P1 epithelial	P1 bone	Adult bone	Adult bone
Primary Palate / Premaxilla	18.4%	23.3%	32.4%	19.7%	24.4%	31.7%	30.7%
Ant. Sec. Palate / Maxilla	45.7%	47.5%	39.9%	49.5%	47.4%	41.8%	42.8%
Post. Sec. Palate / Palatine & Pterygoid	35.9%	29.2%	27.7%	30.8%	28.3%	26.5%	26.5%

## Data Availability

μCT images, limb bud microscopy photographs, landmark coordinate data, and associated morphometric analysis R scripts are available publicly as a dataset within Dryad (https://doi.org/10.5061/dryad.ghx3ffbvb; Welsh et al., 2024).

## Abbreviations:

A-P: anterior-posterior
CNC: cranial neural crest
FNP: frontonasal process
HREM: High resolution episcopic microscopy
MxP: maxillary processes
mnp: medial nasal processes
μCT: micro-computed tomography
RGZ: rugae growth zone
TFs: transcription factors
WISH: Whole mount in situ hybridization

## References

[R1] Adams D.C., Collyer M.L., Kaliontzopoulou A., 2020. geomorph: Software for geometric morphometric analyses. R package version 3.3.1.https://cran.r-project.org/package=geomorph

[R2] Boughner J.C., Wat S., Diewert V.M., Young N.M., Browder L.W., Hallgrímsson B., 2008. Short-faced mice and developmental interactions between the brain and the face. Journal of Anatomy 213, 646–662. 19094181 10.1111/j.1469-7580.2008.00999.x PMC2666134

[R3] Bush J.O., Jiang R., 2012. Palatogenesis: morphogenetic and molecular mechanisms of secondary palate development. Development 139, 231–243. 22186724 10.1242/dev.067082 PMC3243091

[R4] Cignoni P., Callieri M., Corsini M., Dellepiane M., Ganovelli F., Ranzuglia G., 2008. Meshlab: an open-source mesh processing tool., in: Eurographics Italian Chapter Conference. pp. 129–136. 10.2312/LocalChapterEvents/ItalChap/ItalianChapConf2008/129-136

[R5] Collyer M.L., Adams D.C., 2018. RRPP: An R package for fitting linear models to high-dimensional data using residual randomization. Methods in Ecology and Evolution.

[R6] Economou A.D., Ohazama A., Porntaveetus T., Sharpe P.T., Kondo S., Basson M.A., , 2012. Periodic stripe formation by a Turing mechanism operating at growth zones in the mammalian palate. Nat Genet 44, 348–351. 10.1038/ng.1090 22344222 PMC3303118

[R7] Enlow D.H., Bang S., 1965. Growth and remodeling of the human maxilla. Am J Orthod 51, 446–464. 10.1016/0002-9416(65)90242-3 14287831

[R8] Fedorov A., Beichel R., Kalpathy-Cramer J., Finet J., Fillion-Robin J.-C., Pujol S., , 2012. 3D Slicer as an image computing platform for the Quantitative Imaging Network. Magnetic Resonance Imaging 30, 1323–1341. 10.1016/j.mri.2012.05.001 22770690 PMC3466397

[R9] Fish J.L., 2019. Evolvability of the vertebrate craniofacial skeleton. Semin Cell Dev Biol 91, 13–22. 10.1016/j.semcdb.2017.12.004 29248471 PMC5999547

[R10] Geyer S.H., Mohun T.J., Weninger W.J., 2009. Visualizing vertebrate embryos with episcopic 3D imaging techniques. ScientificWorldJournal 9, 1423–1437. 10.1100/tsw.2009.154 20024516 PMC5823209

[R11] Hall J., Jheon A.H., Ealba E.L., Eames B.F., Butcher K.D., Mak S.-S., , 2014. Evolution of a developmental mechanism: Species-specific regulation of the cell cycle and the timing of events during craniofacial osteogenesis. Dev Biol 385, 380–395. 10.1016/j.ydbio.2013.11.011 24262986 PMC3953612

[R12] Hallgrímsson B., Jamniczky H., Young N.M., Rolian C., Parsons T.E., Boughner J.C., , 2009. Deciphering the Palimpsest: Studying the Relationship between Morphological Integration and Phenotypic Covariation. Evolutionary Biology 36, 355–376. 23293400 10.1007/s11692-009-9076-5 PMC3537827

[R13] Hammond N.L., Dixon M.J., 2022. Revisiting the embryogenesis of lip and palate development. Oral Dis 28, 1306–1326. 10.1111/odi.14174 35226783 PMC10234451

[R14] Hauser G., Daponte A., Roberts M., 1989. Palatal rugae. Journal of Anatomy 165, 237–249. 17103618 PMC1256673

[R15] Hilliard S.A., Yu L., Gu S., Zhang Z., Chen Y.P., 2005. Regional regulation of palatal growth and patterning along the anterior–posterior axis in mice. Journal of Anatomy 207, 655–667. 10.1111/j.1469-7580.2005.00474.x 16313398 PMC1571556

[R16] Jayasankar P., Banker A., Bhattacharya A., Gandhi R., Patel N., Parikh S., 2016. Quantitative and qualitative analysis of palatal rugae patterns in Gujarati population: A retrospective, cross-sectional study. J Forensic Dent Sci 8, 126. 10.4103/0975-1475.195110 28123265 PMC5210098

[R17] Kaucka M., Petersen J., Tesarova M., Szarowska B., Kastriti M.E., Xie M., , 2018. Signals from the brain and olfactory epithelium control shaping of the mammalian nasal capsule cartilage. Elife 7, e34465. 10.7554/eLife.34465 29897331 PMC6019068

[R18] Kawasaki M., Kawasaki K., Meguro F., Yamada A., Ishikawa R., Porntaveetus T., , 2018. Lrp4/Wise regulates palatal rugae development through Turing-type reaction-diffusion mechanisms. PLoS One 13, e0204126. 10.1371/journal.pone.0204126 30235284 PMC6147471

[R19] Kouskoura T., Kozlova A., Alexiou M., Blumer S., Zouvelou V., Katsaros C., Chiquet M., Mitsiadis T.A., Graf D., 2013. The Etiology of Cleft Palate Formation in BMP7-Deficient Mice. PLos One 8, e59463. 10.1371/journal.pone.0059463 23516636 PMC3597594

[R20] Kurihara S., Enlow D.H., Rangel R.D., 1980. Remodeling reversals in anterior parts of the human mandible and maxilla. Angle Orthod 50, 98–106. 10.1043/0003-3219(1980)050&lt;0098:RRIAPO&gt;2.0.CO;2 6929173

[R21] Li Q., Ding J., 2007. Gene expression analysis reveals that formation of the mouse anterior secondary palate involves recruitment of cells from the posterior side. Int. J. Dev. Biol. 51, 167–172. 17294368 10.1387/ijdb.062212ql

[R22] Luo W., Yi Y., Jing D., Zhang S., Men Y., Ge W.-P., , 2019. Investigation of Postnatal Craniofacial Bone Development with Tissue Clearing-Based Three-Dimensional Imaging. Stem Cells and Development 28, 1310–1321. 10.1089/scd.2019.0104 31392933 PMC6767869

[R23] Maga A.M., 2016. Postnatal Development of the Craniofacial Skeleton in Male C57BL/6J Mice. J Am Assoc Lab Anim Sci 55, 131–136. 27025802 PMC4783629

[R24] Martinez-Maza C., Rosas A., Nieto-Díaz M., 2013. Postnatal changes in the growth dynamics of the human face revealed from bone modelling patterns. J Anat 223, 228–241. 10.1111/joa.12075 23819603 PMC3972044

[R25] Morris Z.S., Abzhanov A., 2021. Heading for higher ground: Developmental origins and evolutionary diversification of the amniote face. Curr Top Dev Biol 141, 241–277. 10.1016/bs.ctdb.2020.12.003 33602490

[R26] Murray S.A., Morgan J.L., Kane C., Sharma Y., Heffner C.S., Lake J., , 2010. Mouse Gestation Length Is Genetically Determined. PLoS ONE 5, e12418. 10.1371/journal.pone.0012418 20811634 PMC2928290

[R27] Musy M., Flaherty K., Raspopovic J., Robert-Moreno A., Richtsmeier J.T., Sharpe J., 2018. A quantitative method for staging mouse embryos based on limb morphometry. Development 145, 1–7. 10.1242/dev.154856 PMC5963863 29540505

[R28] Okano J., Suzuki S., Shiota K., 2006. Regional heterogeneity in the developing palate: morphological and molecular evidence for normal and abnormal palatogenesis. Congenital Anomalies 46, 49–54. 10.1111/j.1741-4520.2006.00103.x 16732762

[R29] Palmer A.R., Strobeck C., 1986. Fluctuating Asymmetry: Measurement, Analysis, Patterns. Annu. Rev. Ecol. Syst. 17, 391–421. 10.1146/annurev.es.17.110186.002135

[R30] Pantalacci S., Prochazka J., Martin A., Rothova M., Lambert A., Bernard L., , 2008. Patterning of palatal rugae through sequential addition reveals an anterior/posterior boundary in palatal development. BMC Developmental Biology 8, 116. 10.1186/1471-213X-8-116 19087265 PMC2637861

[R31] Pauws E., Hoshino A., Bentley L., Prajapati S., Keller C., Hammond P., , 2009. Tbx22null mice have a submucous cleft palate due to reduced palatal bone formation and also display ankyloglossia and choanal atresia phenotypes. Hum Mol Genet 18, 4171–4179. 10.1093/hmg/ddp368 19648291 PMC2758147

[R32] Percival C.J., Green R., Marcucio R., Hallgrímsson B., 2014. Surface landmark quantification of embryonic mouse craniofacial morphogenesis. BMC Developmental Biology 14, 1–12. 10.1186/1471-213X-14-31 PMC4222779 25059626

[R33] Percival C.J., Liberton D.K., Pardo-Manuel de Villena F., Spritz R., Marcucio R., Hallgrímsson B., 2016. Genetics of murine craniofacial morphology: diallel analysis of the eight founders of the Collaborative Cross. Journal of Anatomy 228, 96–112. 10.1111/joa.12382 26426826 PMC4694168

[R34] Peterkova R., Klepacek I., Peterka M., 1987. Prenatal development of rugae palatinae in mice: scanning electron microscopic and histologic studies. Journal of craniofacial genetics and developmental biology 7, 169–89. 3624420

[R35] R Core Team, 2021. R: A language and environment for statistical computing. R Foundation for Statistical Computing, Vienna, Austria [WWW Document]. URL https://www.R-project.org/

[R36] Sarnat B.G., 1997. Some methods of assessing postnatal craniofaciodental growth: a retrospective of personal research. Cleft Palate Craniofac J 34, 159–172. 10.1597/1545-1569_1997_034_0159_smoapc_2.3.co_2 9138513

[R37] Schneider R.A., 2015. Regulation of Jaw Length During Development, Disease, and Evolution. Curr Top Dev Biol 115, 271–298. 10.1016/bs.ctdb.2015.08.002 26589929

[R38] Selleri L., Rijli F.M., 2023. Shaping faces: genetic and epigenetic control of craniofacial morphogenesis. Nature Reviews Genetics 24, 610–626. 10.1038/s41576-023-00594-w 37095271

[R39] Tamarin A., 1982. The formation of the primitive choanae and the junction of the primary and secondary palates in the mouse. American Journal of Anatomy 165, 319–337. 10.1002/aja.1001650308 7180818

[R40] Tonge C.H., McCance R.A., 1965. Severe undernutrition in growing and adult animals: 15. The mouth, jaws and teeth of pigs. British Journal of Nutrition 19, 361–372. 10.1079/BJN19650034 5891038

[R41] Vora S.R., Camci E.D., Cox T.C., 2015. Postnatal Ontogeny of the Cranial Base and Craniofacial Skeleton in Male C57BL/6J Mice: A Reference Standard for Quantitative Analysis. Front Physiol 6, 417. 10.3389/fphys.2015.00417 26793119 PMC4709510

[R42] Welsh I.C., Feiler M.E., Lipman D., Mormile I., Hansen K., Percival C.J., 2024. Dryad dataset: Palatal segment contributions to midfacial anterior-posterior growth. 10.5061/dryad.ghx3ffbvb PMC12159334 39831750

[R43] Welsh I.C., Hagge-Greenberg A., O’Brien T.P., 2007. A dosage-dependent role for Spry2 in growth and patterning during palate development. Mechanisms of Development 124, 746–761. 10.1016/j.mod.2007.06.007 17693063 PMC2043129

[R44] Welsh I.C., Hart J., Brown J.M., Hansen K., Rocha Marques M., Aho R.J., , 2018. Pbx loss in cranial neural crest, unlike in epithelium, results in cleft palate only and a broader midface. Journal of Anatomy 233, 222–242. 10.1111/joa.12821 29797482 PMC6036936

[R45] Welsh I.C., O’Brien T.P., 2009. Signaling integration in the rugae growth zone directs sequential SHH signaling center formation during the rostral outgrowth of the palate. Developmental Biology 336, 53–67. 10.1016/j.ydbio.2009.09.028 19782673 PMC2789450

[R46] Wilson R., McGuire C., Mohun T., Project DMDD, 2016. Deciphering the mechanisms of developmental disorders: phenotype analysis of embryos from mutant mouse lines. Nucleic Acids Res 44, D855–861. 10.1093/nar/gkv1138 26519470 PMC4702824

[R47] Young N.M., Hu D., Lainoff A.J., Smith F.J., Diaz R., Tucker A.S., , 2014. Embryonic bauplans and the developmental origins of facial diversity and constraint. Development 141, 1059–1063. 10.1242/dev.099994 24550113 PMC3929406

[R48] Yu K., Ornitz D.M., 2011. Histomorphological study of palatal shelf elevation during murine secondary palate formation. Developmental Dynamics 240, 1737–1744. 10.1002/dvdy.22670 21618642 PMC3275090

[R49] Yu L., Gu S., Alappat S., Song Y., Yan M., Zhang X., , 2005. Shox2-deficient mice exhibit a rare type of incomplete clefting of the secondary palate. Development 132, 4397–4406. 10.1242/dev.02013 16141225

[R50] Yuan Y., Loh Y.-H.E., Han X., Feng J., Ho T.-V., He J., , 2020. Spatiotemporal cellular movement and fate decisions during first pharyngeal arch morphogenesis. Sci Adv 6, eabb0119. 10.1126/sciadv.abb0119 33328221 PMC7744069

[R51] Zelditch M., Swiderski D., Sheets D.H., 2012. Geometric morphometrics for biologists: a primer, 2nd ed. Elsevier Academic Press, San Diego.

[R52] Zhao H., Feng J., Ho T.-V., Grimes W., Urata M., Chai Y., 2015. The suture provides a niche for mesenchymal stem cells of craniofacial bones. Nat Cell Biol 17, 386–396. 10.1038/ncb3139 25799059 PMC4380556

