# Supplementary material for: Palatal segment contributions to midfacial growth in three inbred mouse strains
Source: bioRxiv. 2023 Oct 5:2023.10.03.560703. Preprint. [Version 1] doi: 10.1101/2023.10.03.560703 (PMC10592893; doi:10.1101/2023.10.03.560703)
Supplement: Supplementary file 1 [file media-1.pdf]

Supporting Tables and Figures

Supporting Table 1 – Epithelial landmark definitions, collected on the superficial tissue surface of the face and palate of developmental age E11 – E15 specimens, with a subset collected on P1 specimens. See also Fig. 2.

Landmark Number	Epithelial Landmark Definition
**1**	Most rostral midline point at the meeting of the medial nasal processes, becoming the most rostral midline point on the rhinarium.
**2**	The most ventral midline point along the developing lip on the primary palate, in the region that develops into the lip vermillion.
**3(9)**	Dorso-caudal corner of the whisker field, taken on the skin right next to the plateau of the whisker field, rather than on the field itself; taken where the lateral nasal process and forebrain region meet within E11.5 embryos.
**4(10)**	Rostral apex of the forming medial canthus of the eye; not collected at P1 because of eyelids.
**5(11)**	Caudal apex of the forming lateral canthus of the eye, not collected at P1 because of eyelids
**6(12)**	The caudal most point of the intersection between the lateral nasal process and the maxillary process; taken at the edge of the margin between second and third whisker rows, counting from the top in older embryonic specimens.
**7(13)**	Point at the middle of the medial side of the nasal aperture, taken on the medial edge of the nasal aperture at the point of concave inflection between upper and lower portions of the developing nasal aperture.
**8(14)**	The dorsal most point of the nasal aperture.
**15**	Most rostral midline point of the primary palate ruga; may be the same position as LM 16 and 17 in E11.5 embryos.
**16**	Most caudal midline point of the primary palate ruga; may be the same position as LM 15 and 17 in E11.5 embryos
**17**	Most caudal midline point of the primary palate; may be the same position as LM 15 and 16 in E11.5 embryos.
**18(22)**	Rostro-buchal extent of secondary palate, placed at a lateral edge of the secondary palate where it meets the primary palate; may be at the same position as LM 19(23) in E11.5 embryos.
**19(23)**	Rostral-lingual corner of the palatal shelf, placed on the rostro-medial corner of the growing secondary palatal shelf; may be at the same position as LM 18(22) in E11.5 embryos.
**20(24)**	Point on the medial palatal shelf extent, at the border between anterior and posterior secondary palate, taken at the dorsal ventral inflection of the palatal shelf.
**21(25)**	Caudal-lingual corner of the palatal shelf. In later embryonic ages, the point should be taken in proximity to the posterior end of the pterygoid process.

Supporting Table 2 – Skeletal palate landmark definitions collected on upper jaw bones of P1 and adult specimens. See also Fig. 8.

**Epithelial Homolog**	**P1 & Adult Number**	**Definition**
15	S1(S5)	Most rostral point of the superior incisor alveolus, taken at the midline of the incisor. In P1, taken at approximate location of the incisor midline due to incomplete dental development.
18(22)	S2(S6)	Ventral point on the maxilla-premaxillary suture, taken on the premaxilla.
20(24)	S3(S7)	Most caudal point on the molar alveolus, taken on approximately the midline axis of the molar row. In P1, taken at the most caudal point of the developing maxillary alveolus.
21(25)	S4(S8)	Most ventrocaudal point of the pterygoid process.

Supporting Figures

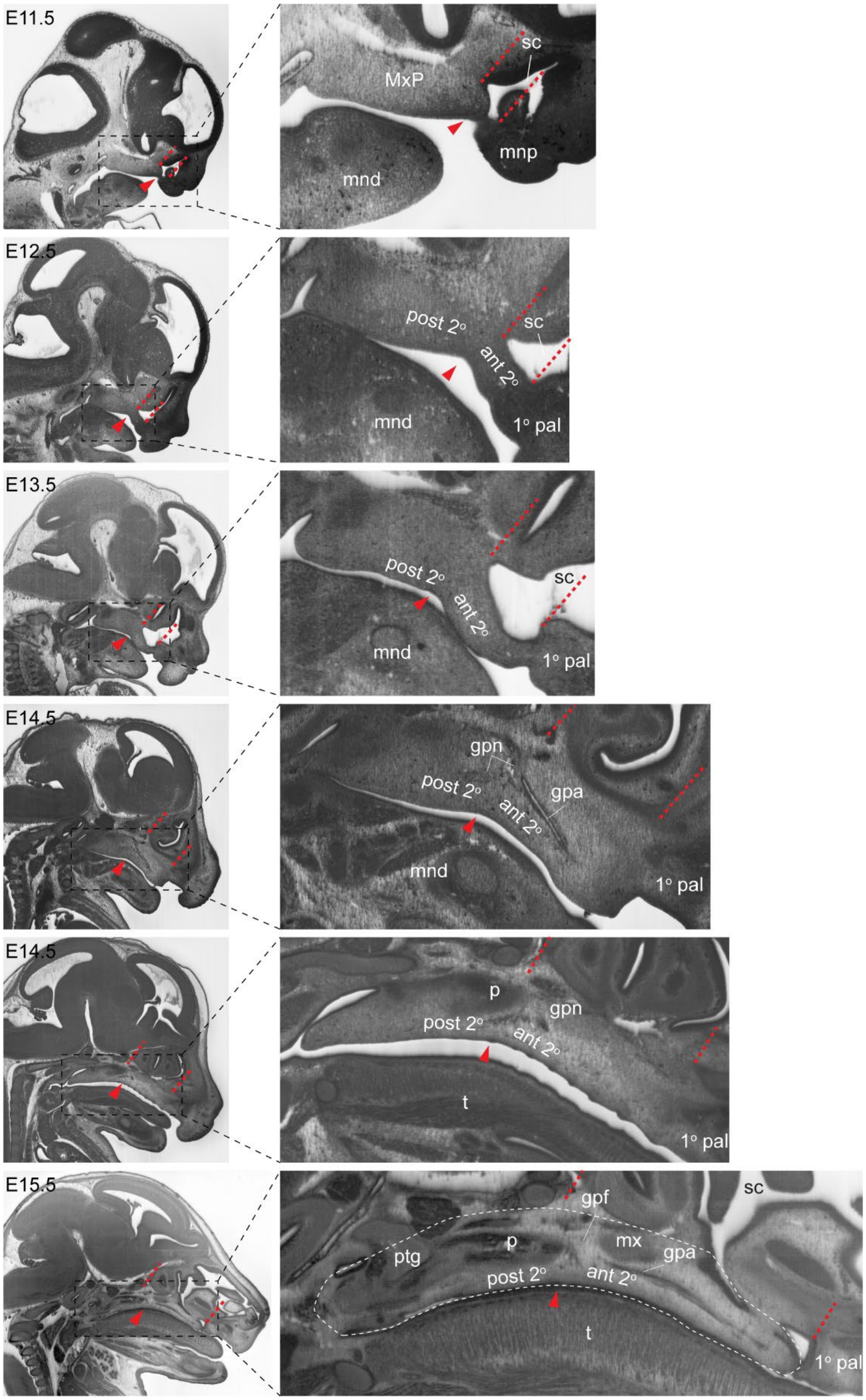

Supporting Figure 1 – Segmental growth dynamics of the midface relative to A-P landmarks – Sagittal sections of wildtype embryos imaged with high resolution episcopic mircroscopy (HREM) provide histological resolution of anatomical landmarks A-P orientation of during midfacial elongation between E11.5 and E15.5. Two E14.5 samples show pre- and post-elevation of the secondary palate. Red arrowhead marks the position of ruga 1, red double headed arrow marks posterior wall of the nasal capsule. White dashed outline demarcates the secondary palate at E15.5. HREM data available at: https://www.ebi.ac.uk/biostudies/bioimages/studies/S-BIAD490?query=DMDD.

Abbreviations: anterior secondary palate (ant 2º), greater palatine artery (gpa), greater palatine foramen (gpf), greater palatine nerve (gpn), mandible (mnd), maxilla (mx), maxillary process (MxP), medial nasal process (mnp), posterior secondary palate (post 2º), primary palate (1º pal), sinus cavity (sc), tongue (t).

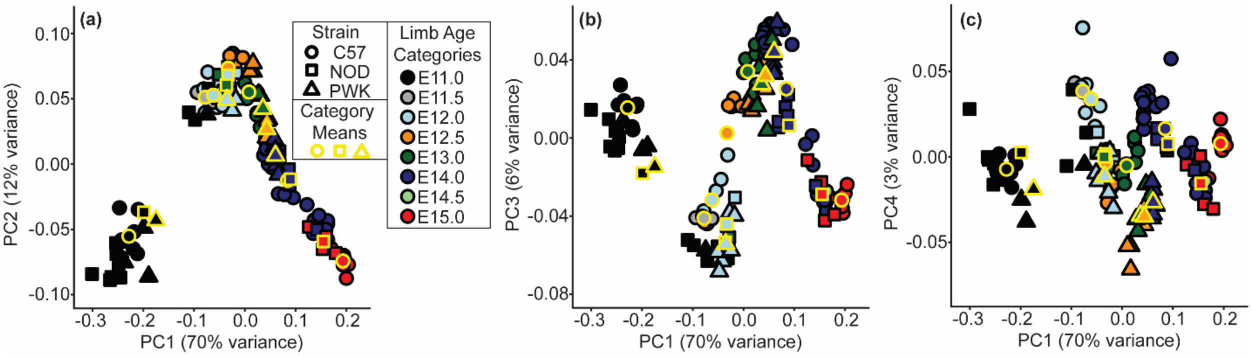

Supporting Figure 2 - Major axes of embryonic facial shape variation across strains – C57 (circle), NOD (square) and PWK (triangle) embryo specimens plotted along the (a) first and second principal component axes of shape (i.e., PC1 and PC2), (b) along PC1 and PC3, and (c) along PC1 and PC4. Circle color indicates the limb based developmental age categories of each specimen, with the yellow bordered circles indicating the mean PC scores for mice of that age category from a single strain. The principal component axes were estimated from the full sample of E11-E15 developmental age embryos, including C57, NOD, and PWK samples. The proportion of facial shape variance associated with each principal component are provided.

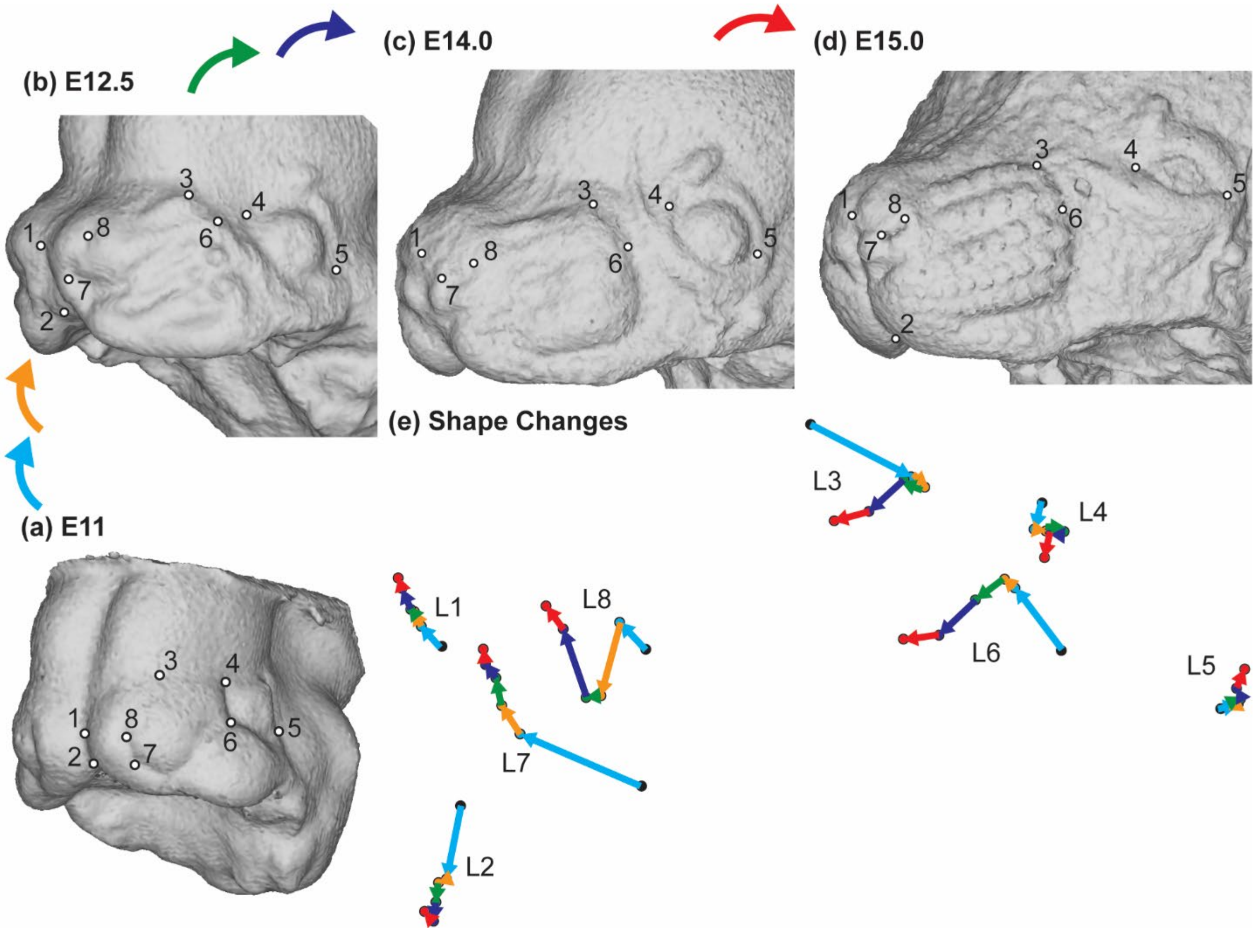

Supporting Figure 3 – C57 facial landmark growth trajectories – The positions of facial landmarks are identified on representative C57 specimens at limb-derived developmental ages (a) E11, (b) E12.5, (c) E14, and (d) E15. E) The average facial landmark positions are plotted for each developmental age category that has more than one C57 sample. These landmark positions represent facial shape after the removal of head scale during Procrustes superimposition. The arrows indicate the trajectory of shape change for each landmark between ages. Black=E11; Light Blue = E12; Orange = E12.5; Green = E13; Dark Blue = E14; Red = E15.

The most noticeable change in facial shape occurred between E11 and E12, as the facial prominences finished fusing and the nasal region began to elongate. During this period, the posterior edge of the whisker field consolidated and was found close to the anterior canthus of the eye. However, the direction of shape vectors changed after E12.5, as the relative distance between the whisker region and eye increased. In addition, changes in shape vectors were noted for two landmarks of the nasal aperture (L7 and L8) as the shape of the nostril remained relatively short and bent with the growth of nearby portions of the

rhinarium. The double shift of specimen PC3 scores across developmental time (Fig. 4B) were partially based on these nasal shape changes (Fig. 10H).

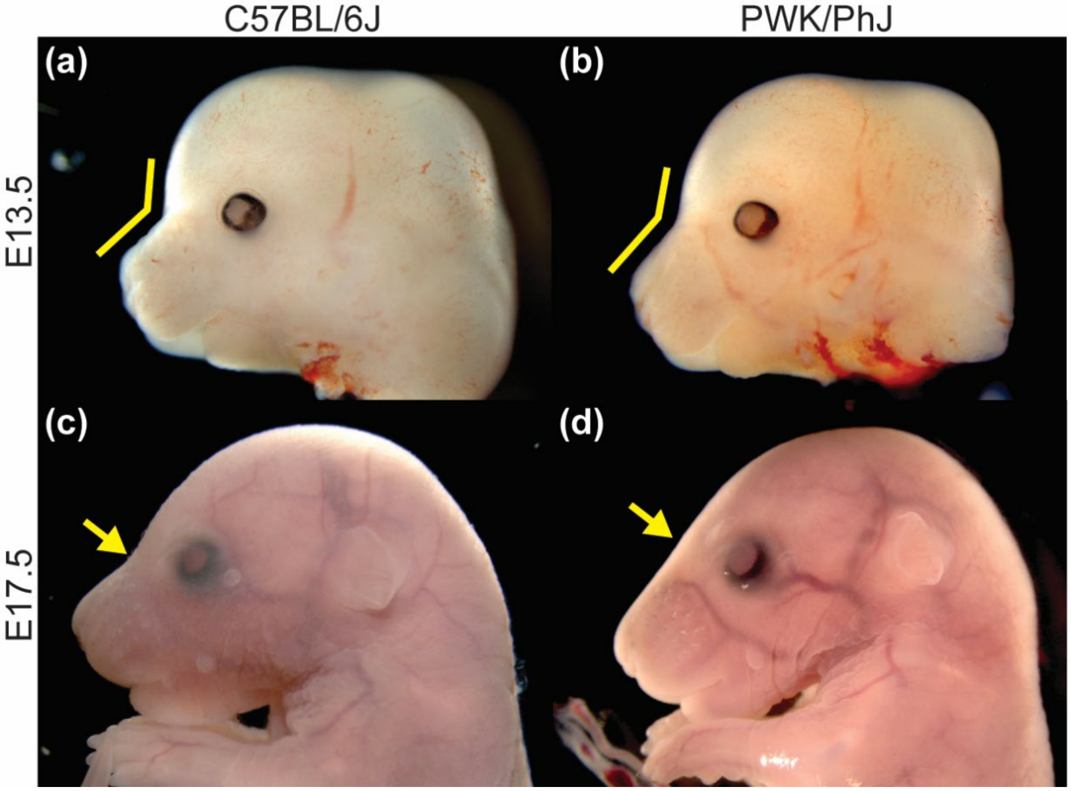

Supporting Fig. 4 – Visualization of nasal/whisker region angle when compared to the rest of the face across strains and ages, with a less wide angle seen within (a, c) C57 specimens when compared to (b, d) PWK specimens, as also suggested by morphometric PC4 shape comparisons (Fig. 10I)
